# Systematic Benchmarking of a Noise‐Tolerant Conductive Hydrogel Electrode for Epidermal Bioelectronics

**DOI:** 10.1002/advs.202515131

**Published:** 2025-11-20

**Authors:** Nazmi Alsaafeen, Ioannis Ziogas, Shirina Alsaedi, Mouza Alshehhi, Shahd Almheiri, Adil Rehman, Khulood Al Shehhi, Hani Saleh, Ahsan Khandoker, Charalampos Pitsalidis, Antoun Khawaja, Anna‐Maria Pappa

**Affiliations:** ^1^ Department of Biomedical Engineering Khalifa University Abu Dhabi 127788 UAE; ^2^ Center for Separations and Catalysis Khalifa University Abu Dhabi 127788 UAE; ^3^ Department of Computer and Information Engineering Khalifa University Abu Dhabi 127788 UAE; ^4^ Department of Physics Khalifa University Abu Dhabi 127788 UAE; ^5^ Healthcare Engineering Innovation Center Khalifa University Abu Dhabi 127788 UAE; ^6^ Advanced Research and Innovation Center (ARIC) Khalifa University Abu Dhabi 127788 UAE; ^7^ Institute of Electronic Structure and Laser (IESL) Foundation for Research and Technology FORTH) N. Plastira 100, Vassilika Vouton Heraklion Crete 70013 Greece; ^8^ Khawaja Medical Technologies GmBH 82041 Munich, Deisenhofen Germany; ^9^ Center for Biotechnology Khalifa University Abu Dhabi 127788 UAE

**Keywords:** conducting hydrogels, conducting polymers, ECG monitoring, electrophysiological recordings, PEDOT:PSS, wearable electronics

## Abstract

Conventional Silver/Silver Chloride (Ag/AgCl) electrodes remain the clinical standard for electrophysiological monitoring but are hindered by poor skin conformity, mechanical rigidity, and signal degradation, particularly under motion or sweat. Here, two hydrogel‐based alternatives are presented and benchmarked using a wireless commercial platform: a porous poly(3,4‐ethylenedioxythiophene):polystyrene sulfonate scaffold infused with hydrogel (PPSCF), and an all‐hydrogel, crosslinker‐free electrode (PPHG) synthesizes via a scalable, one‐pot process. PPHG demonstrates intrinsic stretchability, self‐adhesion, and biocompatibility, forming stable, low‐impedance contacts with skin. Electrochemical measurements reveal that PPHG maintains a capacitive interface with reduced resistive losses, low loss tangent, high dielectric constant, and fast relaxation dynamics, features that enable intrinsic signal smoothing and noise suppression. In a cohort of 39 participants, PPHG electrodes outperform Ag/AgCl in electrocardiography (ECG), showing reduced motion artifacts, higher signal‐to‐noise ratios, and clear preservation of P‐, R‐, and T‐waves. Electroencephalography (EEG) recordings demonstrate enhanced alpha–delta separation, while electrooculography (EOG) and electromyography (EMG) signals exhibit greater amplitude and sharper features. Machine learning analysis of ECG signals reveals a 2.2‐fold improvement in inter‐lead classification accuracy. These findings position PPHG as a soft, adhesive, and sustainable alternative for high‐fidelity, multimodal bioelectronic interfaces, with strong potential for wearables and clinical monitoring systems.

## Graphical abstract

General schematic of the approach.

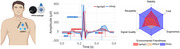

## 1 Introduction

Over the past two decades, skin‐conformal bioelectronic interfaces have become vital tools for noninvasive, real‐time monitoring of physiological signals, enabling both clinical diagnostics and personalized health tracking across various contexts.^[^ ^1^ ^]^ Standard surface recordings, for example electrocardiography (ECG), electromyography (EMG), electrooculography (EOG), and electroencephalography (EEG) typically rely on silver/silver chloride (Ag/AgCl) gel electrodes. While these electrodes offer electrical stability and clinical compatibility, However, the preapplied gel, essential for reducing interface impedance, can dry out over time, notably during extended recordings, resulting in increased impedance and signal degradation. Moreover, the gel may cause skin irritation, and the need for reapplication makes those electrodes inconvenient for long‐term or repeated use.^[^ ^1^, ^2^, ^3^ ^]^ Strategies such as gel preapplication and synthetic adhesives attempt to enhance skin contact but introduce additional issues, including electrolyte drying, sweat‐induced degradation, and skin irritation. These challenges reflect a fundamental mismatch between the soft, ionically conductive biological tissues and the rigid, electron‐conducting materials currently in use.^[^ ^2^, ^4^, ^5^, ^6^ ^]^

Conducting hydrogels have emerged as promising alternatives, combining the ionic conductivity, water content, and softness of hydrogels with the electrical performance of conducting polymers.^[^ ^2^, ^4^, ^7^, ^8^ ^]^ Their intrinsic tissue‐like mechanics and mixed ionic‐electronic conduction enable low‐impedance, motion‐tolerant skin interfaces that preserve signal fidelity under dynamic conditions.^[^ ^5^, ^9^, ^10^, ^11^ ^]^ Recent advances have explored diverse hydrogel architectures incorporating materials such as poly(3,4‐ethylenedioxythiophene):polystyrene sulfonate (PEDOT:PSS), ionic liquids, and nanostructures to enhance electrochemical performance.^[^ ^4^, ^7^, ^10^, ^12^, ^13^ ^]^ PEDOT:PSS‐based scaffolds, for example have been shown to offer excellent electrical properties and enhanced biocompatibility and processability into biomimetic 3D structures,^[^ ^6^, ^14^, ^15^ ^]^ yet their application for electrophysiology remains underexplored.^[^ ^7^, ^16^, ^17^ ^]^

We previously reported a stretchable, biocompatible conducting hydrogel (*Golde*) composed of gelatin, glycerol, chitosan, and can be further doped with a conducting material for example PEDOT:PSS.^[^ ^5^ ^]^ *Golde* exhibited stable ionic and electronic conductivity, inherent adhesiveness, biocompatibility, and thermoreversible behavior, without relying on synthetic toxic crosslinkers, such as glutaraldehyde or ionic solutions, e.g., Ca^2+^. Its soft, water‐rich matrix, maintained reliable performance under strain, humidity, and perspiration, offering mechanical durability and electrical stability over time, suitable for wearable applications.^[^ ^5^ ^]^ Unlike several reported conducting hydrogel electrodes,^[^ ^7^, ^9^, ^18^, ^19^ ^]^ *Golde* can be readily fabricated and stored under ambient conditions, facilitating both its preparation and reuse. Its structural, electrochemical, and mechanical properties have been comprehensively characterized in our previous work.^[^ ^5^ ^]^ Building on these findings, we now investigate the hydrogel's applicability for physiological signal acquisition in real‐world, wearable settings. To this end, we benchmark its performance against the clinical gold standard, the Ambu BlueSensor L electrodes, which are widely regarded as the most reliable gel‐based electrodes for electrophysiological and ECG monitoring. Performance metrics, including signal fidelity, motion artifact resistance, and user comfort, were evaluated across a diverse cohort of 39 individuals to assess practical utility in dynamic, everyday environments. Two configurations of the *Golde* hydrogel electrodes were evaluated: a homogeneous PEDOT:PSS‐doped hydrogel (PPHG), and a composite architecture comprising a PEDOT:PSS scaffold infused within the hydrogel (PPSCF). The better performing configuration, the PPHG was further tested for EMG, EOG, and EEG and for long‐duration ECG recordings to assess comparative performance. PPHG is shown to function as a soft, skin‐conformal interface capable of attenuating high‐frequency noise while preserving low‐frequency bioelectrical signal components with high fidelity. This dual capability is explored here under physiological conditions. Using a commercially available wearable acquisition system (ECG‐ON, Khawaja Medtech, Germany), we conducted three‐lead chest ECG recordings on the 39 participants representing a range of ages and ethnicities. Signal quality was quantitatively assessed using time‐ and frequency‐domain metrics across extended recording sessions. In addition to objective performance evaluations, we collected postrecording feedback to assess user comfort, perceived wearability, and electrode preference, following a protocol adapted from prior studies.^[^ ^7^ ^]^ This dual assessment provides a comprehensive understanding of the hydrogel electrodes’ suitability for real‐world bioelectronic applications.

From a postprocessing perspective, correlating the product of digital waveform properties with a material or substance that acts as a sensing interface holds promise for a wide range of applications in biomedical and mechanical engineering sciences.^[^ ^20^ ^]^ Solving the inverse estimation problem of inferring a material's bioelectrical^[^ ^21^ ^]^ and biomechanical^[^ ^22^ ^]^ properties, or localizing sources of bioelectrical activity,^[^ ^23^ ^]^ largely depends on the recording quality of the sensing material. On this foundation, signal processing and machine learning algorithms facilitate sophisticated and automated prediction of latent variables of interest that drive patterns of variability in the observed phenomenon, whether clinical, chemical,^[^ ^24^ ^]^ mechanical,^[^ ^25^ ^]^ or bioelectrical.^[^ ^26^ ^]^ Our analysis on ECG recordings focuses on an inverse estimation of time and frequency domain landmarks that directly connect to desirable sensing properties. We perform a head‐to‐head comparison of our hydrogel electrode (PPHG) against an Ambu BlueSensor L Ag/AgCl electrodes, which are specifically designed for long‐term recordings as a baseline at scale, to extract robust identifiers that demonstrates the PPHG capabilities over Ag/AgCl as a high‐fidelity filter in terms of sensitivity and noise attenuation. We then link those identifiers to inter‐personal and inter‐lead differences to realize a more comprehensive view. Through detailed frequency and time domain feature extraction, we demonstrate that PPHG provides enhanced frequency response and boosts the expressivity of the ECG landmarks R‐peak, P‐wave, and T‐wave, which are clinically highly valuable in reaching an accurate diagnosis.

## 2 Results and Discussion

### 2.1 Scalable Fabrication and Skin‐Conformable Integration

**Figure 1:**
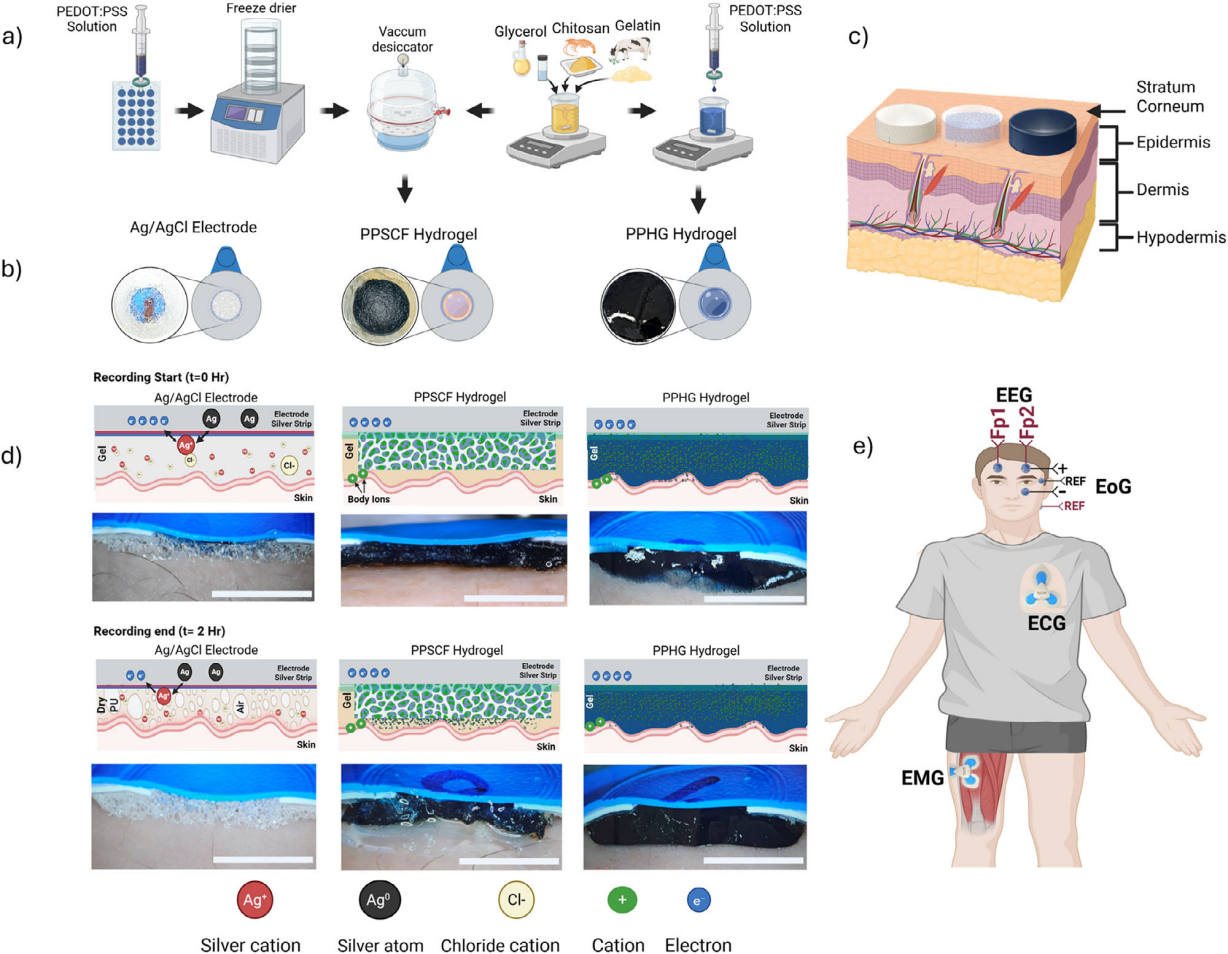
a) Simple one pot synthesis method of hydrogel base material fabrication and it's processing to form the PPSCF and PPHG variants. b) Active electrode area in each variant: Silver/Silver Chloride (Ag/AgCl), PPSCF hydrogel infused electrodes, and entirely hydrogel electrodes (PPHG) with a photograph of the actual electrodes (thickness and diameter were maintained among all three variants 1 mm × 1.7 cm). c) Schematic of human skin layers cross section which illustrates the source capacitive and resistance of the skin. d) Comparison of the mechanism of biosignal transduction in conventional electrodes in comparison to our proposed electrodes, in addition to the emphasis of the degradation in each electrode variant after few hours of recording time. The scale bar is 8.5 mm. e) The body electrophysiological signals that were obtained using our electrodes, portable setup and the location of electrodes placement (forehead for frontal EEG, around the left eye for EOG, Left chest area for ECG, Outer side of the left thigh Muscle group EMG).

To identify the most effective configuration for *Golde*, our self‐adhesive and stretchable hydrogel electrode, we evaluated two design **strategies** (**Figure** **1**a). The first, PPSCF, consists of a porous, freeze‐dried PEDOT:PSS scaffold infused within the hydrogel.^[^ ^2^, ^4^, ^13^, ^27^ ^]^ The second configuration (PPHG) integrates PEDOT:PSS directly into the hydrogel matrix, forming a continuous mixed‐conduction network. The first approach requires a more complex, multistep fabrication process and demonstrated poor mechanical resilience during wear. The hydrogel passively infiltrates the scaffold but fails to form a robust and stable interface, limiting its practicality for long‐term biosignal monitoring. In contrast, the all‐hydrogel design simplifies fabrication into a single step, enhances uniformity, and improves handling and skin contact (Figure 1b; and Figure S1 and Video S1, Supporting Information). PPHG indeed appears to conform to the skin overcoming the high‐impedance barrier to common electrodes that suffer from due to poor mechanical matching with skin (Figure 1c). The Ag/AgCl electrode is shown to have an adequate reliable connection to the skin on a freshly opened Ag/AgCl electrode, however, several gaps at the skin interface begin to show up due to the electrolyte drying over long period of use as shown in the photographs of Figure 1d. In contrast, the hydrogel electrodes, PPSCF and more significantly PPHG depict a conformal interface. These electrodes typically exhibit a Cassie–Baxter type wetting behavior, resulting in increased motion artifacts contrary to the conformable, self‐adhering, high water content materials that appear more consistent with a Wenzel‐type wetting regime.^[^ ^28^, ^29^ ^]^ The signal transduction mechanism in PPSCF and, more prominently, in PPHG differs fundamentally from that of standard Ag/AgCl electrodes, such as the Ambu BlueSensor. While Ag/AgCl electrodes rely on faradaic redox reactions facilitated by hydrated gel electrolytes, this mode of conduction is inherently unstable over time. As the gel dries, impedance rises and signal quality deteriorates, limiting long‐term reliability and user comfort.^[^ ^30^ ^]^ In contrast, PPSCF and PPHG operate via a mixed ionic–electronic conduction mechanism based on volumetric capacitance. This enables the formation of a stable and spatially distributed electrical double layer (EDL) throughout the hydrogel network, promoting efficient signal coupling across the bioelectronic interface.^[^ ^3^, ^28^, ^29^ ^]^ The hydrated, conformal, and self‐adhering nature of these hydrogels not only enhances skin‐electrode adhesion and interface comfort but also functions as a dynamic material‐level filter. This helps maintain low and stable impedance under mechanical deformation, thereby reducing motion artifacts and supporting high signal‐to‐noise ratio (SNR) recordings across multiple electrophysiological modalities, including EOG, frontal EEG, ECG, and surface EMG (sEMG) (Figure 1e; and Figures S1 and S2, Supporting Information).

### 2.2 Electrochemical and Dielectric Performance

Given the predominantly capacitive nature of bioelectric signals and the different conduction mechanisms of PPHG, PPSCF, and Ag/AgCl electrodes, an electrochemical evaluation was performed to identify the most suitable configuration for stable, long‐term epidermal recordings. Key parameters including impedance, capacitance, and electrochemical stability were assessed using electrochemical impedance spectroscopy (EIS) and cyclic voltammetry (CV) across physiologically relevant frequencies.^[^ ^32^, ^33^ ^]^

**Figure 2:**
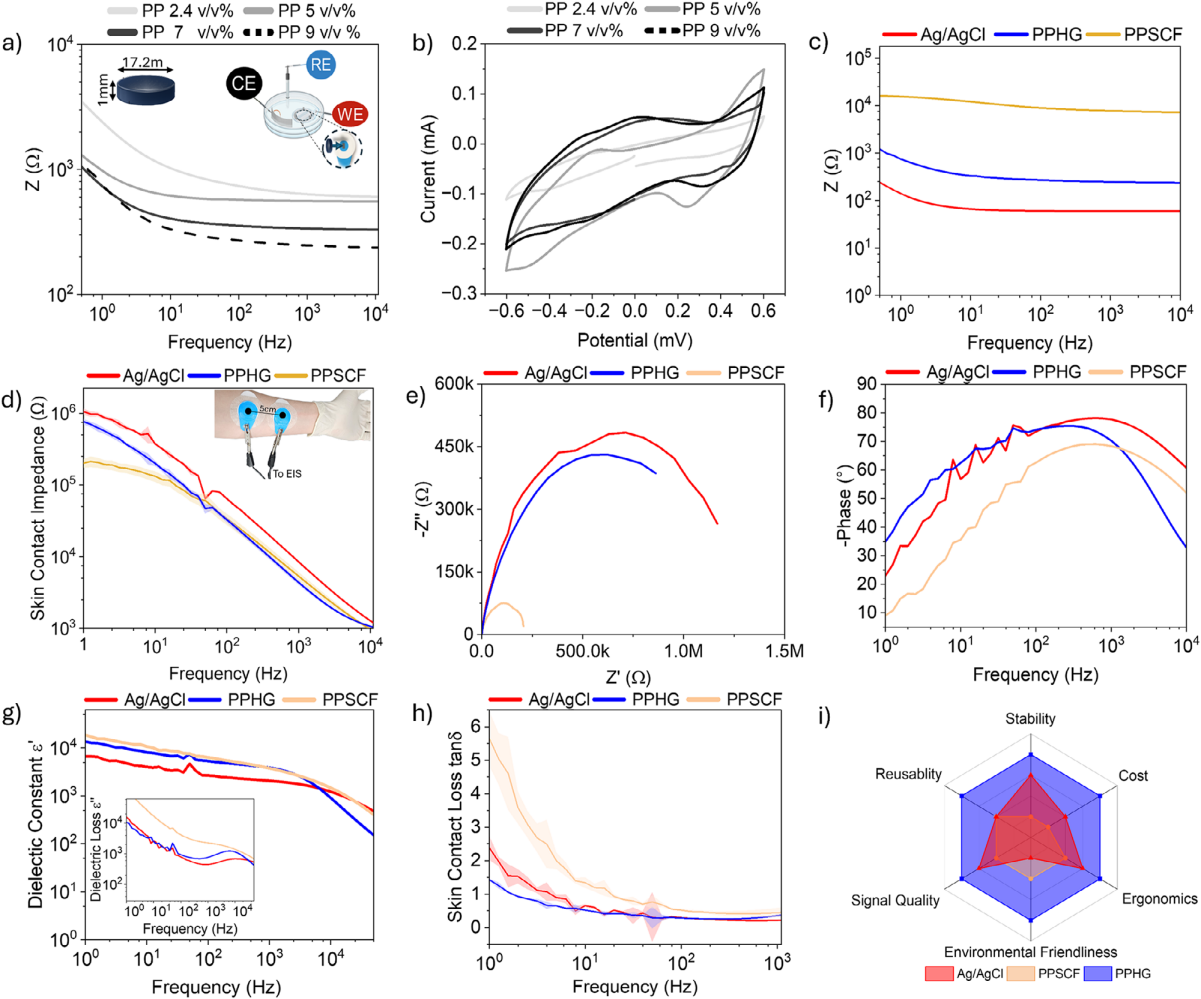
a) Electrochemical impedance spectroscopy (EIS) of hydrogel electrodes with varying PEDOT:PSS doping levels, measured in a PBS setup using a three electrode configuration. b) Cyclic voltammetry of the electrodes in the same setup. c) Bulk impedance (Z) comparison of the optimized formulation (9 v/v% PEDOT:PSS, denoted PPHG) against PPSCF hydrogel and commercial Ag/AgCl electrodes (Ambu) in PBS. d) Skin–contact impedance comparison of PPHG, PPSCF, and Ag/AgCl electrodes. e) Nyquist plots of the three electrode types. f) Phase angle response over the 1 Hz –10 kHz frequency range. g) Dielectric constant and dielectric loss (inset) of the three electrodes across 1 Hz to 10 kHz. h) Relaxation behavior in terms of loss tangent (tan ***δ***). i) Overall qualitative assessment of electrodes’ properties, where a score based system of 1–4 grades similar to this works in the literature,^[^ ^31^ ^]^ assigned 4 = excellent, 3 = very good, 2 = acceptable, and 1 = needs improvement. Furthermore, the scoring justification is discussed in Tables S1–S4 (Supporting Information). Data shown are averages of triplicate samples (*n* = 3) per electrode type. Panels (2d–h) were derived from skin‐contact EIS measurements. 1 Hz to 10 kHz frequency range was used to demonstrate the full behavior of the tested electrodes.

To ensure a fair comparison, the PPHG formulation was first optimized to balance electrochemical performance with mechanical integrity and signal fidelity during ECG acquisition. PEDOT:PSS concentrations of 2.4, 5, 7, 9, and 11 v/v% were tested, with the 9 v/v% showing the most favorable behavior, while the 11 v/v% started exhibiting compromised hydrogel stability and loss of structural integrity under physiological conditions (37 °C). EIS measurements (**Figure** **2**a) showed that increasing PEDOT:PSS content led to a marked reduction in impedance across the full spectrum, with the 9 v/v% variant exhibiting the lowest values, particularly within the 0.5–100 Hz range relevant to electrophysiology. At low frequencies (0.1–10 Hz), all hydrogel variants displayed a capacitive response, transitioning to a more resistive behavior by 100 Hz.^[^ ^3^, ^27^ ^]^ Correspondingly, CV analysis (Figure 2b) revealed symmetrical loops with minimal hysteresis, indicating low resistive losses and good charge reversibility. The 9 v/v% formulation demonstrated the highest charge storage capacity and current response, without evidence of polarization artifacts within the applied potential window (−0.6 to +0.6 V).^[^ ^33^ ^]^

Unlike earlier studies that characterized only the bulk material,^[^ ^5^ ^]^ the electrochemical performance reported here was evaluated on fully assembled electrodes to better reflect operational conditions (Figure 2a; and Figure S3, Supporting Information). The optimized 9 v/v% PEDOT:PSS hydrogel electrode, henceforth termed PPHG, demonstrated superior electrochemical properties suitable for high‐fidelity biosignal acquisition. A practical demonstration of its capacitive behavior is shown in Video S2 (Supporting Information), where the hydrogel is able to power a red LED briefly following disconnection from a DC power source.

To benchmark PPHG against PPSCF and the commercial Ag/AgCl electrodes, bulk impedance was first evaluated using the same setup described in the inset of Figure 2a. As shown in Figure 2c and Figure S4 (Supporting Information), PPHG exhibited a significantly lower impedance, i.e., approximately one order of magnitude lower than PPSCF across the entire frequency spectrum, indicating superior conductivity. At low frequencies (<10 Hz), PPHG displayed a pronounced capacitive response, aligning with the typical frequency range of motion artifacts and baseline drift. This suggests an intrinsic filtering capability, likely driven by strong interfacial polarization and double‐layer charge storage.^[^ ^1^, ^3^, ^8^ ^]^ By contrast, PPSCF showed a flatter, more resistive profile, consistent with limited ionic mobility within the PEDOT:PSS scaffold. Ag/AgCl electrodes, while also exhibiting low‐frequency capacitive behavior, showed overall lower bulk impedance, although this can be attributed to their porous sponge‐like structure filled with ionic gel and their faradaic charge‐transfer mechanism, rather than a true advantage in stable, long‐term bioelectronic interfacing. At higher frequencies (>100 Hz), all three electrode types exhibited similar impedance profiles dominated by resistive behavior, indicative of convergent high‐frequency conduction mechanisms (Figure 2c).

Given the intended application, interface‐level electrochemical performance was further examined to better simulate real‐world conditions. Skin‐contact impedance profiles revealed that Ag/AgCl electrodes exhibited the highest overall contact impedance and the most pronounced signal instability at 10 Hz and in the 50/60 Hz range, commonly associated with motion artifacts, baseline drift, and AC power‐line interference (Figure 2d).^[^ ^27^, ^32^ ^]^ These fluctuations overlap with key signal components, such as the QRS complex in ECG, compromising recording fidelity. In contrast, both PPSCF and PPHG electrodes demonstrated substantially lower distortion across this frequency band. While PPSCF showed slightly lower initial contact impedance than PPHG, both converged by ≈10 Hz, suggesting comparable performance within the relevant bandwidth (1–100 Hz).^[^ ^34^ ^]^

Nyquist analysis of the skin‐electrode interface (Figure 2e) further confirms PPHG's superior capacitive characteristics. PPHG exhibited a broad, well‐defined semicircular arc with a low‐frequency relaxation peak at *f*_max_ ≈1 Hz, indicating high interfacial capacitance, and using the formula below, a slower and more stable relaxation time (*τ* ≈ 0.159 s) and efficient charge transfer that yields a stronger low frequency capacitive coupling. This behavior could be analogous to that of a small, stable ceramic capacitor that is optimized for low‐noise, high‐fidelity signal transduction

(1) $$\tau =\frac{1}{2\pi f_{\max}}$$

In contrast, Ag/AgCl electrodes showed a larger, more distorted semicircle with a relaxation peak at *f*_max_ ≈4 Hz, suggesting higher charge‐transfer resistance, faster relaxation time (*τ* ≈ 0.0398s) and weaker low‐frequency charge retention and less effective dielectric filtering and thus lower coupling and lower frequencies biosignals acquisition efficiency. This profile resembles an electrolytic capacitor that is larger but more prone to leakage and environmental sensitivity, indicative of its less stable and interference‐prone interface. PPSCF, although displaying lower contact impedance initially, presented the smallest semicircle with a relaxation peak at *f*_max_ ≈10 Hz, indicating the fastest relaxation time (*τ* ≈ 0.0159s) which is consistent with minimal capacitive behavior and primarily resistive conduction. These findings likely result from weak interfacial polarization and limited ionic mobility between the hydrogel and the scaffold network, resulting in reduced filtering capability and signal stability.

PPSCF, although displaying lower contact impedance initially, presented the smallest semicircle with a relaxation peak at ≈10 Hz, consistent with minimal capacitive behavior and primarily resistive conduction. These findings likely stem from weak interfacial polarization and limited ionic mobility between the hydrogel and the scaffold network, resulting in reduced filtering capability and signal stability.

These trends were consistent with phase angle behavior across frequency (Figure 2f). At ≈1 Hz, PPHG exhibited a phase angle of ≈−35°, closest to the ideal capacitive phase (−90°), followed by a smooth transition and broad plateau between 100 Hz and 1 kHz. This steady response suggests persistent capacitive dominance, strong interfacial polarization, and robust skin coupling essential for effective suppression of external interference. Ag/AgCl electrodes, by comparison, showed an initial phase angle of −23° and a delayed, less stable capacitive rise, consistent with their faradaic redox mechanism and reliance on hydrated electrolytes.^[^ ^35^ ^]^ PPSCF exhibited the weakest capacitive characteristics, with an initial phase angle of −10°, the farthest from the ideal −90° which is expected for a purely capacitive system. Its slower phase development, likely delayed plateau formation, and early decline beyond 1 kHz further indicating poor capacitive coupling to the skin and elevated resistive losses due to limited ionic transport within the scaffold. These phase response characteristics (Figure 2f) align with observations from the skin‐contact impedance and Nyquist analyses (Figure 2d–e) of the three electrode types.

To elucidate the intrinsic filtering behavior and capacitive dynamics of each electrode, dielectric characterization was conducted. Loss tangent (tan *δ*)—a key metric reflecting dielectric energy dissipation due to dipolar relaxation and ionic drag—was used to evaluate how well each electrode preserves signal integrity under electromagnetic perturbation. Since tan *δ* is defined as the ratio of dielectric loss (*ε*″) to dielectric constant (*ε*′), both parameters were measured and are presented in Figure 2g. As shown in Figure 2g, PPHG and PPSCF displayed similarly high dielectric constants relative to Ag/AgCl, consistent with ionic conduction modes observed in biological tissues.^[^ ^6^ ^]^ In particular the Ag/AgCl electrode displayed a lower *ε*′ and showed perturbations around power line frequencies (50/60 Hz), indicating reduced dielectric stability. Notably, PPHG exhibited the lowest dielectric loss (*ε*″) and followed a frequency‐dependent trend analogous to Ag/AgCl, which has been historically optimized for electrophysiological recordings in the 1–100 Hz range. PPSCF, in contrast, showed the highest dielectric loss, reflecting inferior dipolar alignment and greater signal dissipation. This combination of low dielectric loss and high permittivity promotes efficient alignment of dipoles and mobile ions with weak bioelectric fields, particularly at low frequencies (<1 kHz) where most electrophysiological signals reside. Together, this dielectric behavior confers an intrinsic low‐pass filtering effect, selectively preserving low‐frequency signal components, such as ECG P and T waves or EMG tonic/phasic elements, while attenuating high‐frequency noise. This enables clearer characterization of diagnostically relevant parameters, such as muscle tone, contraction strength, and motor unit recruitment, supporting applications in cardiology, rehabilitation, and neuromuscular diagnostics.^[^ ^36^ ^]^ A demonstration of PPHG's capacitive energy storage is shown in Video S2 (Supporting Information), where a red LED remains lit for several seconds after disconnecting a DC power source.

From a materials standpoint, these findings can be explained by the fundamental structural and functional differences among the electrode types. Ag/AgCl electrodes, composed of a silver‐coated strip in contact with a porous sponge saturated with electrolyte, are nonpolarizable and electrochemically stable. However, they are prone to interfacial buildup, irreversible faradaic reactions, and electrolyte drying, limiting their long‐term performance and driving their common single‐use recommendation.^[^ ^30^, ^37^ ^]^ In contrast, both PPSCF and particularly PPHG overcome these limitations through material‐level design. Glycerol, a core component of *Golde*, modulates dielectric properties by influencing hydrogen bonding, water retention, and polymer mobility. These effects impact the dielectric constant and ionic relaxation dynamics, contributing to a more stable capacitive coupling and reduced dielectric losses in hydrated systems. As previously reported,^[^ ^36^ ^]^ glycerol enhances polymer chain mobility and promotes more efficient ionic conduction by acting as a plasticizer, thereby facilitating a more favorable and stable dielectric response. This ensures long‐term stability, resilience to ambient noise, and reliable performance under practical wearable bioelectronic conditions. Cumulatively, PPHG demonstrated the most favorable electrochemical and dielectric profile, characterized by low skin‐contact impedance, low loss tangent (tan *δ*), stable capacitive response, and tunable material behavior through glycerol inclusion. These properties are essential for reliable, noise‐tolerant, and sensitive signal acquisition in epidermal bioelectronics.^[^ ^36^ ^]^

### 2.3 Multiparametric Comparison

Beyond electrochemical performance, Figure 2i provides a multiparametric comparison, highlighting additional advantages of PPHG. The scoring is based on an unweighted Pugh matrix, with each parameter supported by experimental results, literature estimates, participant feedback from the cohort study, or qualitative assessments of environmental impact. Further details are provided in Table S1 of the Supporting Information. PPHG distinguishes itself as a sustainable alternative to Ag/AgCl electrodes, particularly through its life‐cycle advantages in composition, fabrication, and end‐of‐life management (Figure S5, Supporting Information). All components are biocompatible and can be responsibly processed or disposed of after use. A summarized overview of the sustainability of these constituents, including sourcing, processing, and disposal considerations, is provided in Table S2 (Supporting Information).^[^ ^38^, ^39^, ^40^, ^41^, ^42^, ^43^, ^44^, ^45^ ^]^ PPHG is fabricated in low‐temperature (<70 °C), water‐based process without toxic crosslinkers, minimizing both energy input and hazardous waste. At end‐of‐life, the natural polymer components, readily dissolve and are digested by microorganisms.^[^ ^46^ ^]^ While PEDOT:PSS is not inherently degradable, its low concentration (≈9 v/v%) and incorporation within a biodegradable polymer matrix facilitate partial eco‐degradation through microbial or biological processes, such as ingestion by superworms or other microorganisms. Studies have shown that PEDOT:PSS films can undergo benign breakdown in soil and moist environments, through ingestion by superworms, or under controlled thermal treatment.^[^ ^47^ ^]^ Nacre‐inspired PEDOT:PSS/MMT composites have further demonstrated partial biodegradability and microbial consumption.^[^ ^48^ ^]^

Cost efficiency also favors PPHG, as its constituent materials are abundant and the fabrication process is low‐cost. While Ag/AgCl electrodes benefit from industrial‐scale optimization, PPSCF remains cost‐prohibitive due to the high price of PEDOT:PSS and the need for specialized processing equipment (see Tables S3 and S4 for cost estimations, Supporting Information). Reusability is another key advantage, with PPHG maintaining signal fidelity across multiple recording sessions. Ergonomic evaluation further confirmed superior comfort and skin compatibility (Figure S1b–d, Supporting Information), and the signal quality discussed in later sections supports its suitability for long‐term use. Collectively, these attributes identify PPHG as the most practical and reliable candidate among the tested electrodes for sustained, high‐fidelity epidermal monitoring, including ECG acquisition in the forthcoming cohort study.

### 2.4 Multifaceted PPHG Characterization: Stability, Reusability, Environmental Friendliness

In terms of stability, PPHG retains its performance over time under ambient storage conditions (Figures S6–S9, Supporting Information). This durability may be further supported by the antimicrobial properties of chitosan, which may help suppress biofouling (Figure S9, Supporting Information). To assess the stability of the signal quality upon repeated use, ECG recordings were performed on the same subject across five separate sessions. The signal‐to‐noise ratio (SNR) values showed minimal variation, except for the third recording, after which the SNR returned to the initial levels (Figure S7, Supporting Information). We attribute this deviation to suboptimal electrode placement rather than material degradation, as lead III consistently exhibited the lowest SNR among the three ECG leads. As shown in Figure S8 (Supporting Information), coefficient of variation (CoV) analysis was used to quantify the intrinsic repeatability of each electrode type, independent of inter‐sample error. Across the frequency range of 0.5–100 Hz, PPHG electrodes exhibited highly stable impedance values, with CoV below 10% for both fresh and reused samples. In contrast, Ag/AgCl electrodes displayed much greater variability, exceeding 50%–60% at low frequencies, reflecting their sensitivity to hydration state and interfacial polarization. The negligible difference between fresh and reused PPHG electrodes indicates that repeated use does not compromise performance, underscoring the hydrogel's stability and suitability for continuous or long‐term electrophysiological monitoring.

Mechanical durability was also evaluated under cyclic strain (Figure S6, Supporting Information). Although electrochemical impedance spectroscopy (EIS) results are presented in impedance (Ω), sheet conductivity (mS m^−1^) is also reported to enable comparison with other conductive hydrogels. At 10% strain, no significant change in conductivity was observed after 100 stretch–release cycles (mean ± SD: 161.2 ± 21.2 → 182.4 ± 36.5 mS m^−1^; *p* = 0.239), confirming stability under physiological deformation. At 50% strain, a modest but statistically significant decrease (≈18%) was detected (205.7 ± 49.1 → 168.2 ± 24.0 mS m^−1^; *p* = 0.031). Despite this, both conductivity and mechanical hysteresis remained consistent, demonstrating robust electromechanical integrity across 10%–50% cyclic strain. SEM imaging (Figure S9a, Supporting Information) revealed only minor surface deformation and contamination after use, while microbial swab tests (Figure S9b, Supporting Information) showed no bacterial growth on either fresh or used PPHG samples after 48 h of incubation, confirming the material's suitability for reuse.

Collectively, these results demonstrate that PPHG combines biocompatibility, reusability, and sustainability within a single, scalable platform. Its bio‐derived composition enables environmentally responsible fabrication and end‐of‐life disposal, while the low PEDOT:PSS content allows partial biodegradation under natural or microbial conditions.^[^ ^47^, ^48^ ^]^ The one‐pot, low‐temperature, water‐based synthesis further minimizes energy consumption and eliminates toxic by‐products, aligning with principles of sustainable manufacturing. PPHG maintains both mechanical and electrical stability under cyclic strain, retains high signal fidelity after repeated use, and shows no microbial contamination even after extended storage and incubation, as verified by surface swab analysis (Figure S9b, Supporting Information). These features, together with user comfort, inherent adhesion, and stable impedance performance, highlight the material's suitability for long‐term epidermal monitoring and large‐scale physiological studies.

### 2.5 Multimodal Electrophysiological Recording: EEG, EMG, EOG

To demonstrate the signal fidelity and versatility of the PPHG electrode and benchmark it versus the standard, we conducted proof‐of‐concept recordings across three modalities, EOG, EMG, and EEG from a single participant.

**Figure 3:**
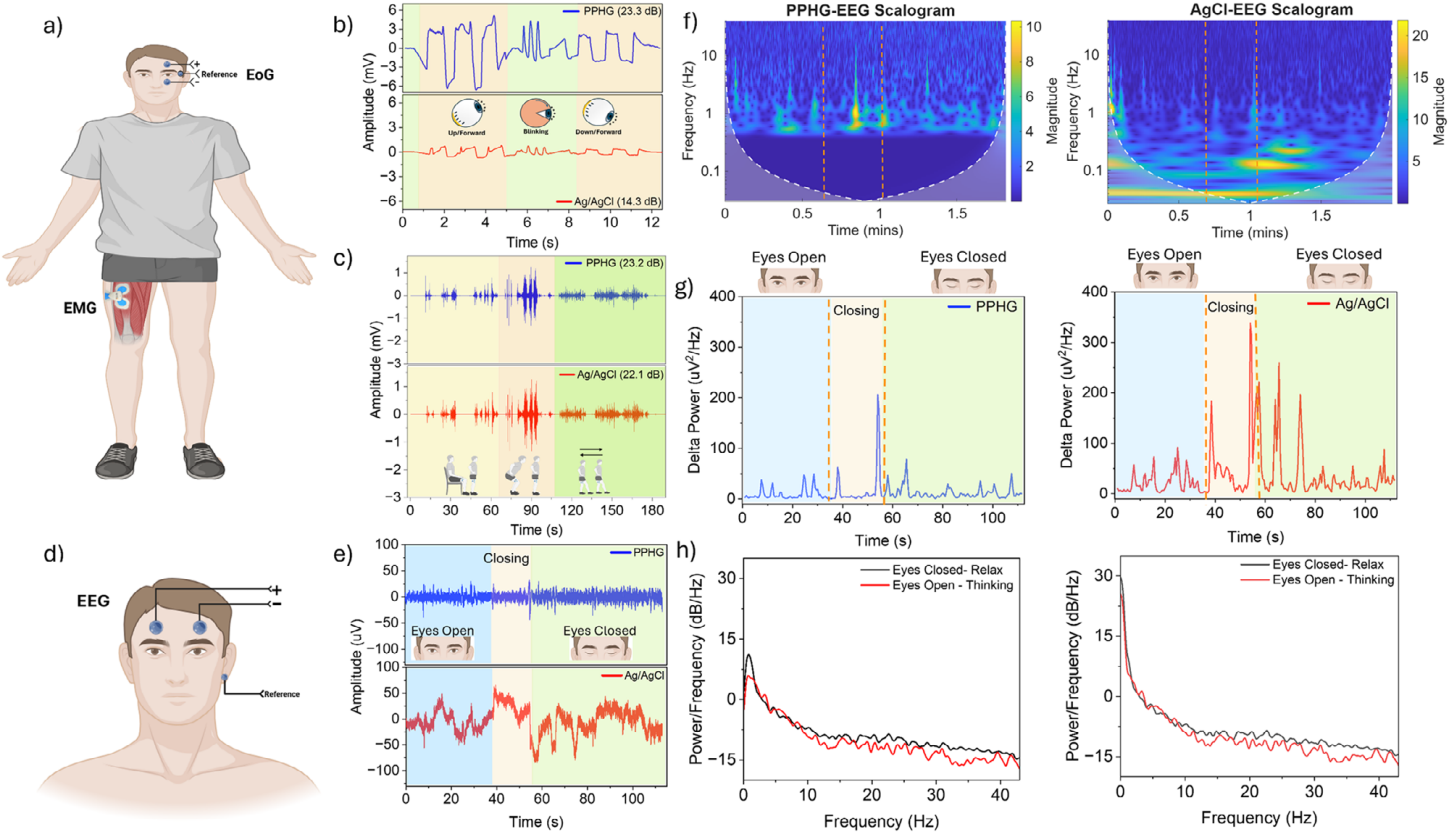
a) Schematic illustration of electrode placement for EOG (left eye) and EMG (left quadriceps femoris muscle group) recordings. b) Representative EOG signals recorded sequentially using conventional Ag/AgCl (red) and hydrogel‐based PPHG (blue) electrodes during ocular activities including upward gaze, triple blinking, and downward gaze. Corresponding signal‐to‐noise ratio (SNR) values for each electrode are indicated. c) EMG signals captured from the quadriceps during a series of motor tasks—sitting, standing, squatting, walking forward, and walking backward—comparing Ag/AgCl (red) and PPHG (blue) electrodes, with respective SNR values shown. d) Electrode placement schematic for frontal EEG recordings at Fp1 and Fp2 with reference behind the right ear. e) Continuous frontal EEG recordings during resting state, depicting eyes open with cognitive engagement (mental arithmetic) followed by eyes closing and eyes closed relaxed states, for Ag/AgCl (red) and PPHG (blue) electrodes. f) Scalograms (continuous wavelet transforms) of EEG signals recorded with PPHG (left) and Ag/AgCl (right) electrodes, illustrating time‐frequency dynamics during resting conditions. g) Time‐resolved delta power (0.5–4 Hz) for EEG signals from PPHG (left) and Ag/AgCl (right), highlighting transitions between eyes open, closing, and eyes closed states. h) Power spectral density (PSD) plots comparing delta band activity during eyes closed relaxation (black) and eyes open thinking (red) states for PPHG (left) and Ag/AgCl (right) electrodes. In panels (f–h), plots on the left correspond to PPHG recordings, and those on the right correspond to Ag/AgCl recordings.

**1) EOG**: As shown in **Figures** **3**a and S11a (Supporting Information), PPHG electrodes were placed near the eyes to record characteristic EOG signals during upward gaze, blinking, and downward gaze. Figure 3b compares the resulting signals from PPHG and conventional Ag/AgCl electrodes. Across all tasks, PPHG captured larger amplitude signals with sharper, more defined peaks, reflecting improved sensitivity to corneo‐retinal and ocular biopotentials.

Quantitative assessment via signal‐to‐noise ratio (SNR) further supports these findings: PPHG achieved an SNR of 23.3 dB, ≈1.5× higher than that of Ag/AgCl (14.3 dB), indicating superior signal clarity. Complementary results in Figure S11a (Supporting Information) show PPHG consistently delivering ≈2× higher peak‐to‐peak amplitudes, even during downward gaze, where signal amplitudes are typically attenuated. Figure S12 (Supporting Information) confirms that PPHG achieves higher SNR across all activities, reinforcing its capacity for clean, high‐fidelity EOG acquisition with reduced motion artifacts.

**2) EMG**: Surface EMG recordings were obtained from the left quadriceps femoris during controlled motor tasks, including sitting, standing, squatting, and walking (Figure 3a; and Figure S11b, Supporting Information). Both PPHG and Ag/AgCl electrodes successfully captured baseline tonic activity and dynamic phasic bursts (Figure 3c). Notably, PPHG demonstrated more pronounced phasic activation during the initial sitting phase, suggesting enhanced responsiveness to subtle muscle contractions.

Across all activities, the PPHG electrode maintained an average SNR of 23.2 dB, modestly higher than the 22.1 dB recorded with Ag/AgCl. While the amplitude profiles were largely comparable, this slight but consistent improvement highlights PPHG's effectiveness in rejecting noise and maintaining signal integrity, supporting its use in dynamic EMG applications.

**3) EEG**: EEG signals were recorded using frontal placements (Fp1 and Fp2) under resting conditions: eyes open with mental activity, eyes closing transition, and eyes closed in a relaxed state (Figure 3d; and Figure S11c, Supporting Information). Despite identical preprocessing, Ag/AgCl signals exhibited residual baseline noise and amplitude instability, whereas PPHG recordings appeared cleaner and more stable (Figure 3e).

Time–frequency analysis using continuous wavelet transform (CWT) revealed clearer spectral features in PPHG recordings (Figure 3f). During mental activity, the PPHG electrode resolved delta (0.5–4 Hz) and theta (7–8 Hz) band activity, while Ag/AgCl signals showed nonspecific low‐frequency power (<0.1 Hz), likely due to slow drift or motion artifacts. During the transition to eyes closed, PPHG captured distinct delta, theta, and alpha (8–12 Hz) oscillations, consistent with resting‐state neural shifts. In contrast, Ag/AgCl recordings lacked band‐specific resolution. While Figure 3g shows that Ag/AgCl recorded higher absolute delta power, Figure 3h demonstrates that PPHG more clearly differentiates delta activity between eyes‐open and eyes‐closed states. This suggests that while Ag/AgCl captures more background low‐frequency power, PPHG more accurately resolves state‐dependent neural oscillations, likely due to better artifact suppression and lower impedance variability.

### 2.6 PPHG Hydrogel Electrode as a High‐Fidelity Filter for Enhanced ECG Quality

**Figure 4:**
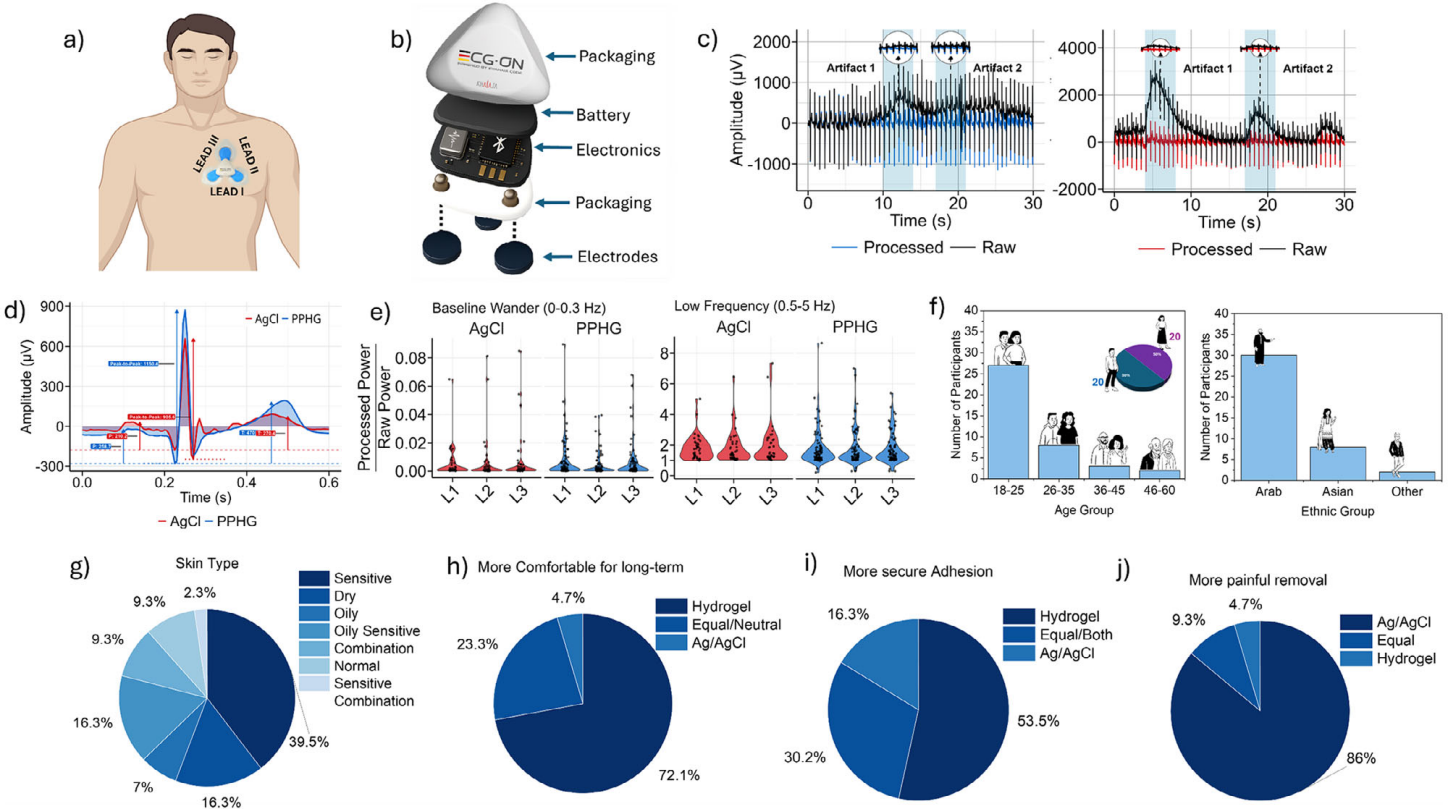
a) ECG signal recording placement location for the cohort study participants and the three lead electrode system used. b) Illustration of the portable ECG holter device used. c) Manifestation of moving artifacts (highlighted in blue shaded intervals) in the PPHG sensor (left panel) and AgCl sensor (right panel) of participant p5. d) Representative heartbeat centroid of a subject (p10) from manually cleaned lead I recording (centroid waveform obtained from the clustering analysis presented in Figure 5. e) Processed‐to‐raw power ratio of the ECG in frequency ranges related to noise and ECG content, for the entirety of the available data. f) The diversity of the cohort study population age and ethnicity. (g) Collection of the different skin types ranging from dry, oily, and sensitive. h–j) The cohort study feedback on the comfort for long term recording, secure connection to the body, and the pain experienced upon the removal of the electrode.

To evaluate the performance of PPHG electrodes in electrocardiography (ECG), we recorded signals using a wireless Holter monitor (ECG.ON) configured with a 3‐lead system placed over the left chest (**Figure** **4**a,b). The PPHG electrodes were reused on a subset of participants without degradation in signal quality or adverse skin effects, supporting their potential for safe and effective reusability, which is detailed in the multiparametric comparison, reusability section. We assessed ECG quality through both time‐ and frequency‐domain analyses, focusing on motion artifacts and key clinical waveform features.

In the time domain (Figure 4c), PPHG electrodes exhibited reduced baseline wandering compared to Ag/AgCl, indicating natural suppression of motion‐induced noise. This attenuation was consistent across all three leads (Figures S2 and S13, Supporting Information), reducing the need for aggressive postprocessing and preserving signal fidelity.

Beyond noise suppression, PPHG electrodes enhanced the visibility of ECG landmarks, including the P‐wave, T‐wave, and R‐peak amplitude (Figure 4d). These features are critical for beat‐to‐beat analysis in clinical and pathological assessments, where subtle morphological changes, particularly in the P and T waves, are associated with cardiovascular conditions, such as ischemic stroke, hypertrophic cardiomyopathy, and therapy response. This emergent PPHG trait designates a favorable clinical and pathological alternative to the widely used Ag/AgCl, as it allows for enhanced characterization and feature extraction in beat‐to‐beat ECG computerized analysis, that is the prognostic standard in several clinical and pathological conditions detected from the ECG. These can manifest as slight morphological alterations on the P‐wave^[^ ^49^, ^50^, ^51^ ^]^ and T‐wave^[^ ^52^, ^53^, ^54^ ^]^ landmarks.

Frequency‐domain analysis further supports these findings (Figure 4e). Following standardized ECG preprocessing (including baseline wandering motion artifact rejection, low‐pass filtering, band‐stop filtering for removing powerline noise and its harmonics and bad data segments rejection by means of impedance and variance thresholds, see the Experimental Section), we compared the raw and cleaned power spectral densities (PSD) across frequency bands. PPHG recordings more closely resembled the cleaned PSD than Ag/AgCl, indicating lower intrinsic noise and minimal filtering distortion. In low‐frequency bands associated with motion artifacts (Figure 4e, left panel), PPHG exhibited higher processed‐to‐raw power ratios, suggesting that the signal was less impacted by baseline drift, validating its role as a material‐level filter (Figures S13 and S14, Supporting Information). In mid‐ and high‐frequency bands including LF, MF, HF, and UHF2, PPHG maintained power ratios near unity (Figure S13b–e,i, Supporting Information), indicating high spectral integrity. Ag/AgCl showed slightly greater stability in the first UHF range (Figure S13g, Supporting Information), but no statistically significant differences were found across comparisons.

Signal‐to‐noise ratio (SNR) values across leads I–III were comparable between the two electrode types. Ag/AgCl marginally outperformed in Lead I (20.28 vs 19.17 dB), while PPHG recorded higher SNR in Lead II (14.82 vs 14.30 dB) and Lead III (11.38 vs 10.06 dB). While absolute SNR values were lower than our previous report,^[^ ^5^ ^]^ this reflects more conservative noise definitions and less controlled acquisition environments, rather than inconsistencies in electrode performance. It is worth noting that these differences do not contradict prior findings but instead reflect how the SNR value is sensitive to both electrode–skin interface characteristics and the specific analysis/estimation method. Notably, the PPHG electrode maintained comparable signal fidelity across all leads, reinforcing its robustness and practical equivalence even superiority across several aspects, to standard Ag/AgCl electrodes in real‐world conditions.

We attribute the intrinsic low‐pass filtering of PPHG to be primarily originating from its volumetric/interfacial capacitance. In a hydrated PEDOT:PSS hydrogel, ionic, and electronic carriers interact within a 3D network, forming distributed electrical double layers that relax slowly. This produces long dielectric relaxation times, so low‐frequency bioelectric signals are preserved while higher‐frequency noise and motion artifacts are naturally attenuated. Our experiments support this mechanism: impedance and dielectric analyses show that PPHG exhibits the highest capacitance, highest dielectric constant, and lowest dielectric loss tangent compared to Ag/AgCl and PPSCF controls. These features indicate efficient capacitive coupling rather than resistive damping. Controlled comparisons, including PEDOT:PSS composition sweeps, homogeneous PPHG versus scaffolded PPSCF, and bulk versus skin‐contact spectra, consistently show that interfacial/volumetric capacitance and dielectric relaxation dominate, with ionic mobility (stabilized by hydrogel hydration and glycerol) setting the characteristic time constant. The inherent PPHG adhesiveness supports conformable and intimate interface with the skin. This maintains a stable and consistent contact and minimizes electrode–skin separation, thereby preserving interfacial capacitance, while glycerol helps keep the interface moist and reliable over time. In summary, the low‐pass effect is an intrinsic material property of the hydrated PEDOT:PSS/hydrogel network, not a measurement artifact, and is reproducibly confirmed across controlled experiments.

### 2.7 Participants Feedback

Chitosan, gelatin, and glycerol are well‐established biomaterials widely used in wound dressings and implantable devices, and are generally recognized for their biocompatibility. Conductive polymers, particularly PEDOT:PSS, have also demonstrated favorable cytocompatibility in both in vitro and in vivo studies.^[^ ^46^ ^]^ Commercial PEDOT‐based coatings such as Amplicoat have passed ISO 10 993 biological evaluations for long‐term electrophysiology, neuromodulation, and cardiac rhythm management applications.^[^ ^55^, ^56^ ^]^ As expected the biocompatibility of the formulation was confirmed using an almar blue cell proliferation assay for 7 days (Figure S10, Supporting Information).

To simulate real‐world wearable use, we evaluated the mechanical stability and adhesion of the PPHG hydrogel electrode during dynamic conditions. When applied as a circular patch on the hand, the PPHG maintained conformal adhesion and mechanical integrity throughout repeated hand opening–closing cycles (Figure S1a and Video S1, Supporting Information). Furthermore, long‐term wear tests demonstrated that the electrode remained structurally stable after 2, 4, and 14 h of continuous use (Figure S1b, Supporting Information), supporting its robustness for extended electrophysiological monitoring. Feedback was collected from all 39 participants, balanced by sex, and diverse in age, ethnicity, and skin types (Figure 4f,g). Responses indicated that the PPHG electrodes offered superior comfort, stronger adhesion, and lower skin irritation compared to conventional Ag/AgCl electrodes. As shown in Figure 4h–j, 72.1% of participants expressed a preference for PPHG in long‐term use, and 86% reported reduced discomfort during removal. Skin compatibility was further assessed through direct visual comparison of electrode sites. In both hairy and shaven male participants, Ag/AgCl electrodes caused visible redness and minor irritation postremoval, likely due to body hair disruption or adhesive trauma (Figure S1c,d, Supporting Information). In contrast, the PPHG electrodes caused no observable irritation during or after wear, even across multiple hours (Figure S1b, Supporting Information), demonstrating enhanced biocompatibility and suitability for diverse skin types, including sensitive or hair‐bearing regions. Together, these findings highlight the PPHG electrode's potential as a comfortable, robust, and skin‐friendly alternative for long‐term wearable electrophysiological monitoring in varied real‐world scenarios.

### 2.8 Unsupervised Machine Learning‐Driven Inverse Estimation of Heartbeat Profiles Reveals Enhanced ECG Information Allocation Across Leads in PPHG

**Figure 5:**
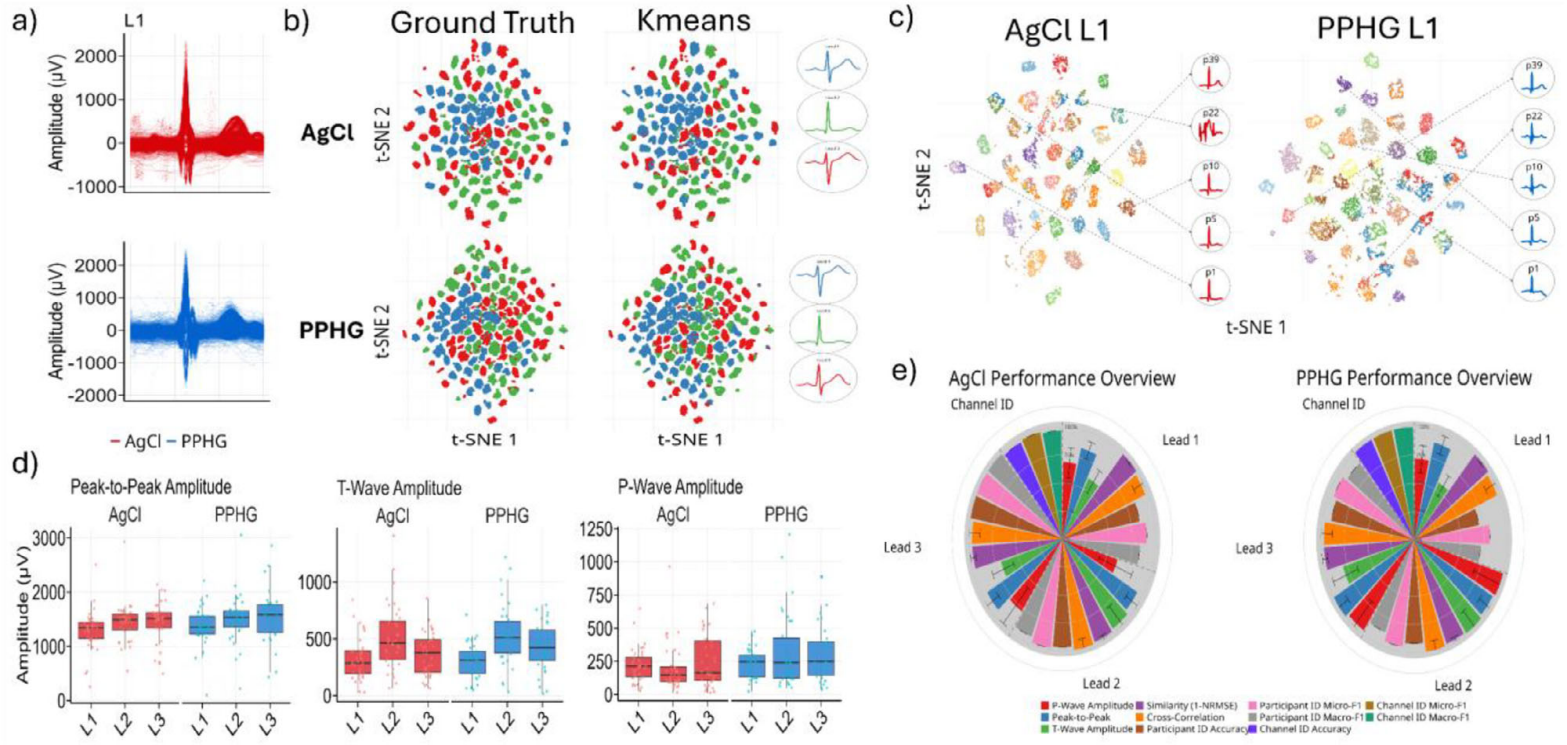
Large‐scale unsupervised clustering analysis to assess signal quality in AgCl and PPHG sensors. a) Superimposed heartbeat waveforms from Lead I (L1) of AgCl and PPHG recordings of 39 participants. (Heartbeats are then projected through Principal Component Analysis (PCA) in a lower‐dimensionality space.) b) *t*‐SNE projection of PCA‐reduced heartbeats, colored by: ground truth lead (left column), identified cluster through *k*‐means (right column). Characteristic waveforms for each lead of every sensor are obtained by applying the inverse PCA transformation on the *k*‐means cluster centroids. c) *t*‐SNE projection of the PCA‐reduced heartbeats, colored by participant for Lead I (L1) (left: AgCl, right: PPHG). A characteristic heartbeat is obtained for each participant by applying the inverse PCA on the cluster centroids. d) ECG morphology features calculated on the participant characteristic centroids, showing slightly elevated sensitivity of the PPHG expressed through increased amplitude. e) Overview of signal quality metrics calculated on participant and lead characteristic centroid heartbeats, assessing ECG morphology, signal similarity (NRMSE and cross‐correlation), and lead/participant identification accuracy through the unsupervised *k*‐means clustering.

In conjunction with the fine‐grained time and frequency‐domain analysis of the PPHG properties in Figure 4, we proceed to examine the large‐scale properties of the PPHG recordings in terms of quality and informativeness. To facilitate a comprehensive machine learning analysis, we detect and analyze the ECG recording at the individual heartbeat level. Using a forced mass R‐peak alignment strategy, we construct batches of heartbeats per participant and per lead. The per lead overlaid heartbeats, shown in **Figure** **5**a, amplify the inter‐personal variability that is expressed bilaterally relative to the R peak. Intra‐personal variability (Figure S17, Supporting Information), although less pronounced, also manifests on the P, Q, S, and T waves, due to the R‐peak‐based alignment. Structuring the data as such is beneficial for uncovering patterns in the data that relate to morphological quality features. Following, the force‐aligned heartbeat data from 39 participants are reduced to a lower‐dimensional manifold through Principal Component Analysis (PCA),^[^ ^57^ ^]^ by retaining 99% of the variance in the original high‐dimensional heartbeat space. We then apply the *k*‐Means clustering algorithm on the PCA‐reduced space to cluster the heartbeats into well‐delimited modes. First, we perform this experiment by setting the number of expected modes to three, corresponding to the ECG leads used for recording. We visualize the clustering performance by projecting the PCA‐reduced space and the *k*‐means identified centroids through the *t*‐distributed Stochastic Neighborhood Embedding (*t*‐SNE)^[^ ^58^ ^]^ manifold learning algorithm. The results demonstrate slightly increased capacity of the PPHG‐derived heartbeats in organizing into clearly separated clusters, compared to AgCl (Figure 5b). To quantify the performance of the unsupervised clustering in the lead identification task, we resort to solving the optimization problem of assigning clusters to their most probable corresponding ground truth classes using the Hungarian or Munkres assignment algorithm.^[^ ^59^ ^]^ We then measure accuracy score, macro‐f1 and micro‐f1 scores on the ground truth and mapped classes, with PPHG (Figure S15a, Supporting Information) surpassing AgCl (Figure S16b, Supporting Information) in the lead identification task in all cases (PPHG ‐ Accuracy: 0.762, Micro‐F1 Score: 0.865/AgCl: Accuracy: 0.74, Micro‐F1 Score: 0.851).

Taking the inverse PCA‐transform of the *k*‐means centroids, we can revert our analysis to the original heartbeat space (Figure S16, Supporting Information), thus obtaining a meaningful illustration of a representative per lead heartbeat profile from the entirety of the dataset with a “confidence” level of ≈85% as dictated by our unsupervised clustering analysis. We observe slightly increased amplitude of PPHG T‐wave compared to AgCl in Lead I (L1), whereas the opposite is evident in Lead II (L2). The R‐peak of the PPHG is more pronounced in Lead II (L2). These results are directly associated with lead identification performance; moreover, their clinical interpretation can also be related to electrode placement, hinting that PPHG is more robust to slight sensor location variability across different individuals and recordings. Furthermore, it can imply greater sensitivity of each PPHG lead to specific ECG components, leading to a lesser overlap of information between leads.

### 2.9 PPHG Demonstrates Reduced Susceptibility to Inter‐Personal Variability as Revealed by Unsupervised Participant Oriented Clustering Analysis

We repeat the unsupervised machine learning analysis through PCA, *k*‐means clustering and solving the cluster assignment optimization problem on the force‐aligned heartbeat data, this time separately for each lead of each sensor, in order to extract conclusions regarding participant‐resonant properties. Our results (Figure 5c; and Figure S17, Supporting Information), reveal superior participant identification performance of the AgCl sensor in all three leads (e.g., Lead II (L2) – AgCl ‐Accuracy: 0.756 Macro‐F1 Score: 0.861 / PPHG – Accuracy: 0.718 Macro‐F1 Score: 0.836). Interpretation of this result may unfold on the basis of AgCl preserving “sequence‐level” features and ignoring the fine‐grained intra‐personal variabilities that manifest within the sequence. On the other hand, our previous results have consolidated the enhanced sensitivity of PPHG and thus interpretation can follow an increased “intra‐participant” beat‐to‐beat variability that hinders identity detection considering the absence of longitudinal recordings for each participant. This manifests through a clustering into modes that have less correspondence to the ground truth of participant identity, and perhaps greater correspondence to specific heartbeat morphologies.

The inverse PCA transform allows us to obtain the characteristic heartbeat profile of each participant from the corresponding mapped centroids, with a reduced confidence of ≈75%. This reduced confidence leads to heartbeat centroids that do not resemble a heartbeat (Figure 5c)—an example is p22 in AgCl Lead I (L1), where the mode mixing leads to heartbeats from different participants being clustered together and yielding an irregular waveform that does not resemble a heartbeat. Utilizing these per‐participant centroid profiles, we perform a morphological quantification analysis and measure P‐wave, peak‐to‐peak and T‐wave amplitudes. Our results (Figure 5d) indicate increased median amplitude for all three measures across all three leads in the PPHG sensor compared to AgCl. Although this effect is not deemed statistically significant, it hints toward an observable tendency of the distributions of these features. Additionally, we measure the similarity of the waveforms of the two sensors per participant and per lead, by means of cross‐correlation and normalized root mean square error (NRMSE). The results shown by Figure S18 (Supporting Information), validate a clear morphological similarity between the two sensors, yet a non‐negligible NRMSE hints toward structural differences in the morphology of the obtained ECG waveforms. In general, we systematically compared PPHG and AgCl using a comprehensive unsupervised machine learning analysis with an orientation toward evaluation of large‐scale lead and participant‐related morphological properties. Figure 5e, summarizes this effort by showcasing an overview of the metric values used; AgCl demonstrated superior participant identification performance, whereas PPHG featured increased lead identification performance, and more pronounced P‐wave, T‐wave, and peak‐to‐peak amplitude across all leads.

## 3 Conclusions

This study systematically benchmarks a crosslinker‐free, single‐step conducting hydrogel electrode designed to address long‐standing limitations of traditional gel‐based systems, particularly Ag/AgCl AMBU electrodes, the prevailing clinical standard for electrophysiological monitoring. The PPHG hydrogel, composed of gelatin, chitosan, and glycerol doped with PEDOT:PSS, combines conformal skin adhesion, mechanical resilience, and stable conductivity without the use of harsh adhesives or ionic gels. This formulation enables low‐noise, motion‐tolerant signal acquisition across multiple bioelectric modalities. Comprehensive electrochemical and dielectric characterization demonstrates that PPHG exhibits lower skin–electrode impedance, improved phase stability, and favorable dielectric behavior relative to commercial electrodes. Its low loss tangent and rapid relaxation time contribute to effective low‐pass filtering, reducing baseline drift and motion artifacts. The hydrogel also maintains mechanical and electrical stability under physiologic strain and repeated use, supported by the antimicrobial activity of chitosan. Benchmarking across ECG, EEG, EMG, and EOG recordings revealed higher signal‐to‐noise ratios and improved waveform fidelity, including clearer P‐ and T‐waves, distinct alpha–delta separation, and robust detection of ocular and muscular activity under dynamic conditions. These results were corroborated by AI‐assisted analysis on a 39‐participant cohort, showing greater robustness to sensor displacement, higher inter‐lead efficiency, and enhanced morphological detail. Although AMBU electrodes retained a slight advantage in certain biometric classification tasks, PPHG captured finer intra‐individual variations, underscoring its relevance for personalized and longitudinal monitoring. Participant feedback also confirmed superior comfort and skin compatibility.

Overall, PPHG represents a biocompatible, reusable, and sustainable hydrogel electrode that combines stable electrical performance with user comfort, providing a viable alternative to conventional Ag/AgCl systems for next‐generation wearable and clinical bioelectronics.

## 4 Experimental Section

Gelatin (bovine skin, Type B, G9391, U.S.), Chitosan (high molecular weight, deacetylated chitin, 419 419, Ireland), acetic acid (100%), glycerol (99%, Fisher Scientific), ethylene glycol (EG), 4‐dodecylbenze nesulfonic acid (DBSA), and (3‐glycidyloxypropyl)trimethoxysilane (GOPS) were obtained from Sigma‐Aldrich. Aqueous PEDOT:PSS dispersion (Clevios PH 1000, 1 L, Germany) was obtained from Heraeus GmbH. Dulbecco's phosphate‐buffered saline (DPBS, Gibco) serving as the release medium. All reagents were used as without further purification. The fabrication and characterization processes were performed using various analytical instruments. Scaffold structures were prepared using SP Virtis Advantage Pro freeze dryer.

### Hydrogel Electrodes Fabrication–Hydrogel Base (Golde) Fabrication

Building upon the method reported by Alsaafeen et al.,^[^ ^5^ ^]^ the stretchable and biocompatible hydrogel matrix (Golde) was synthesized using a straightforward one‐pot approach. Briefly, 12 mL of deionized water was poured into a 50 mL borosilicate beaker and heated to 55 °C. Under continuous stirring with a PTFE‐coated magnetic stir bar (initially at 250 rpm), 3 mL of analytical‐grade glycerol (99%, Fisher Scientific) was added, followed by 3 mL of a pre‐prepared 0.5 wt% high molecular weight chitosan solution. After brief mixing (≈5 min), 3.75 g of bovine‐derived gelatin (Sigma‐Aldrich) slowly introduced to the solution while gradually increasing the stirring speed to 350 rpm to minimize clumping. The reaction was allowed to proceed for 20 min or until the mixture became fully homogeneous and visually golden amber in color. This protocol was scalable, provided that all component ratios were maintained.

### Conducting Polymer Solution Preparation

First, 10 mL of an aqueous dispersion of PEDOT:PSS (Clevios PH1000, Heraeus, Germany) was transferred into a sterile Falcon tube and sonicated for 5 min in an ice‐cold water ultrasonicator, then filtered using a 0.45 µm nylon syringe filter(s). 5 vol% ethylene glycol (EG), 0.5 vol% 4‐dodecylbenzenesulfonic acid (DBSA), and 1 vol% (3‐glycidyloxypropyl)trimethoxysilane (GOPS) were sequentially added, with 5‐min sonication intervals between each addition using an ice‐cold ultrasonic bath. The final mixture was passed through a 0.45 µm nylon syringe filter to remove aggregates. GOPS was either added immediately before integration with the hydrogel matrix or omitted entirely to prevent premature crosslinking that could hinder uniform distribution within the hydrogel network.

### PPHG Hydrogel Electrode Fabrication

To create the homogenous conductive PPHG electrode, the prepared hydrogel was poured into a 10 mL beaker with 5 mL hydrogel solution and 0.5 mL of filtered PEDOT:PSS solution (or depending on the doping concentration of interest) added dropwise, while the hydrogel solution was heated at 55 °C and vigorously stirred for 5 min and degassed to remove any suspended bubbles. The blue PPHG solution was finally poured into the circular molds of 1.8 cm diameter and ≈1.5–2 mm thick (basically filing the mold with 1 mL of PPHG solution).

### PPSCF Hydrogel Electrode Fabrication

PEDOT:PSS scaffolds were synthesized to achieve a porous structure. A 10 mL solution of PEDOT:PSS was sonicated for 10 min, followed by the addition of 0.05 g of DBSA, with 5‐min sonication. To enhance scaffold stability, 0.3 g of GOPS was incorporated, and the solution was sonicated for another 5 min before undergoing magnetic stirring for 30 min to ensure uniform dispersion. 1 mL per well was poured into each of a 6 × 4 well plate the 24‐well plate was then freeze‐dried for 24 h to produce porous scaffolds, which were subsequently baked at 80 °C for 5 h. The dried scaffolds were sliced and are half immersed into the hydrogel solution and placed into a vacuum desiccator for 3 min, this forces the air out of the pores and replaces it with hydrogel, the chamber is then vented to pop any of the bubbles now on the top of the scaffolds, the scaffolds are flipped and the process is repeated until the scaffold is filled with hydrogel.

### ECG Electrodes Assembly with the Hydrogels

To create the final electrode for characterization and electrophysiological recording. Ambu BlueSensor L ECG Ag/AgCl patches are intricately striped of its gel and sponge without impacting the metal strip, using a cotton swab immersed in ethanol the metal strip is swiped 3 times to ensure its clear of any AgCl gel residue. These ready hydrogel electrodes PPHG and PPSCF are placed over the metal strip and are subject to hot air for a second to make it melt and form a complete connection to the metal strip without any air bubbles between it and the metal strip.

### Electrochemical Characterization

To observe the EIS and CV setup refers to Figure S2 (Supporting Information),^[^ ^60^ ^]^ the final ECG electrode for each variant was used as the working electrode (diameter of 1.72 cm), a stainless‐steel rectangular mesh with twice the area of the working electrode was used (1.5 cm × 3.5 cm) and a Glass rod AgCl reference electrode was used as the third electrode. The active electrode was the ECG electrode, the counter electrode a stainless‐steel mesh (twice the active area), and a standard Ag/AgCl electrode served as reference. Each electrode variant was measured three times (*n* = 3) to minimize sample‐to‐sample variation.

### Electrochemical Impedance Spectroscopy (Bode, Phase Aangle, Nyquist Plots)

EIS measurements were conducted on all three electrodes using a potentiostat/galvanostat system (AutoLab PGSTAT204, Metrohm, Sweden). A 10 mV sinusoidal signal was applied across a frequency range of 0.1 Hz to 100 kHz, with particular emphasis on the 1 Hz to 100 kHz window for analysis. Nyquist plot of the three electrodes’ means were plotted and smoothened by an averaging filter to improve their readability and interpretation. The phase angle values were plotted from the EIS data “‐Phase” values means.

### Cyclic Voltammetry

CV was performed on all three electrodes at a scan rate of 0.1 V s^−1^ over a potential range from −0.6 to +0.6 V to avoid hydrolysis caused distortions yet get an accurate representation of the typical electrophysiological amplitudes.

### Skin Contact Impedance

A pair of electrodes is placed 5 cm apart on a human forearm and connected to the (AutoLab PGSTAT204, Metrohm, Sweden) in a 2‐electrode setup (working and counter). In a potentiometric mode an applied sinusoidal signal of 1 mV in amplitude was applied across a frequency range of 0.1 Hz to 100 kHz, similar to work reported.^[^ ^32^ ^]^

### Dielectric Behavior Assessment

Dielectric parameters, such as permittivity (*ε*′, *ε*″), electric modulus (*M*′, *M*″), and loss tangent (tan *δ*) were estimated from the EIS files of each electrode triplicate, based on the algorithms and formulas.^[^ ^36^ ^]^ The peak relaxation frequency Fmax) was the frequency taken at the highest complex impedance (‐Z″). All the measurements were done in triplicates (*n* = 3) and results reported in these plots are the means and standard deviations as error bars.

### Stability and Reusability Assessments

To probe the effect of cyclic loading and environmental degradation on the performance of the electrodes, three tests were performed. Skin contact impedance of the Ag/AgCl electrodes and PPHG was measured for two conditions fresh and dry/used. The same protocol and data (for the fresh samples) from the skin contact impedance test was used.

To ensure that the electrical properties of the hydrogel electrode remain stable under repeated deformation, three dumbbell‐shaped PPHG samples (15 mm × 8 mm × 2 mm thickness) were subjected to cyclic tensile loading and recovery (100 cycles, ≈0.3 Hz, 25 °C) using a Biotester 5000 system (CellScale, Canada). The potential strain experienced by wearable or chest‐mounted electrodes depends on the device geometry, but literature reports typical respiratory strains ranging from ≤1% during normal breathing up to ≈16% under deep or pathological respiration.^[^ ^61^, ^62^, ^63^ ^]^ Accordingly, cyclic tests were conducted at 10% and 50% strain to represent physiologic chest expansion, high‐deformation regimes corresponding to large joint or body movements and engineering durability, respectively. To have a measure of the device durability and considering the apparatus limitations 50% and 70% strains were preliminary test for cycles, where the 70% strain sample experienced significant fatigue and fractured by the ninth cycle establishing it as the upper bound limit of the device. Hence 10% and 50 % strains were adopted. The sample dimensions were chosen based on the mechanical grip and displacement constraints of the test apparatus. Sheet resistance and electrical conductivity (Ossilla, UK; four‐point probe) were measured before and after 100 cycles to evaluate any changes in electrical performance.

Furthermore, SEM images of PPHG electrodes surface was taken to examine changes in the surface morphology or potential microbial growth (if any), fresh PPHG electrodes and overused old and stored PPHG electrodes were placed in a desiccator overnight (this was done to ensure that the samples do not vent any water or air that might be trapped into the vacuum of the SEM). On the next day, these samples were gold coated and SEM images of the surface were taken using the JOEL SEM electron microscope. To further investigate the antibacterial behavior and reusability of the electrodes, two complementary experiments were conducted.

### Surface Bacteria Swab Test

First, an agar plate test was performed to assess possible bacterial contamination after repeated electrode use. Swabs were collected from three electrodes before use (0 uses) and three electrodes after six uses, and the samples were incubated for 24 and 48 h. No bacterial growth was observed on any of the plates. Although it was initially expected to detect some bacterial colonies after six uses, this absence of growth is likely due to the fact that the skin surface was wiped with alcohol prior to each application. As a result, the electrodes were always placed on a clean surface, preventing bacterial transfer or accumulation. These findings confirm that the electrodes can be safely reused, supporting the manuscript's claims regarding their reusability and hygienic use.

### Electrophysiological Signals Recordings

The recording device (ECG‐ON, Khawaja Medical Technology GmBH, Germany) was used to record all the electrophysiological signals, along with the ECG‐ON application for device control and signal recording. The Ambu BlueSensor L electrode backing was used to interface the PPHG electrode with the device (with the pre‐existing AgCl gel filled sponge removed and the silver strip cleaned up).

### EOG Signal Preprocessing and Analysis

Vertical EOG signals were recorded from the left eye (as depicted in Figure 3a) using both conventional Ag/AgCl and hydrogel‐based PPHG electrodes during a single session. Signals were sampled at 200 Hz, while the subject performed controlled ocular tasks including upward gaze, triple blinking, and downward gaze. This full sequence of activities was executed consecutively three times to generate three independent data sets (Set 1, Set 2, and Set 3) for each Ag/AgCl and PPHG. Raw signals were preprocessed using a fourth‐order Butterworth bandpass filter (0.1–15 Hz) to attenuate baseline drift and high‐frequency noise including suppression of powerline interference. Baseline correction was performed by subtracting the signal mean, and detrending was applied to remove residual slow drifts. To further enhance signal quality, wavelet denoising was conducted using a Daubechies 4 (db4’) wavelet at level 3, complemented by a 50 ms median filter to reduce impulsive noise while preserving sharp ocular features.

The cleaned signals were segmented into predefined activity intervals and further divided into overlapping 1‐s epochs with 50% overlap for feature extraction. Peak detection of positive and negative deflections was performed, particularly within the blink segment, to robustly identify ocular events. Peak‐to‐peak (PtP) amplitudes were computed as the difference between maximum and minimum values within 400 ms windows centered on detected peaks.

Signal‐to‐noise ratio (SNR) was estimated for each activity by calculating the variance ratio between the activity segment and a baseline noise segment selected from a quiet period between blinking and downward gaze (≈8.5–8.9 s). For each electrode type (AgCl and PPHG), a total of nine SNR values were extracted: three activities (Look Up, Blink 3x, Look Down) × three repeated sets. SNR values were expressed in decibels (dB) and calculated as

(2) $$SNR_{\mathrm{dB}\;}=10\mathrm{lo}g_{10\;}\left(\frac{\sigma_{\mathrm{signal}}^{2}}{\sigma_{\mathrm{noise}}^{2}}\right)$$

where $\sigma_{\mathrm{signal}}^{2}$ and $\sigma_{\mathrm{noise}}^{2}\;$ denote variances of the activity and noise segments, respectively.

Descriptive statistics of PtP amplitudes and SNR values were visualized using bar plots with error bars indicating standard deviations. This approach enabled a clear comparison of signal quality and ocular event characteristics between the two electrode types without formal hypothesis testing.

To ensure performance and consistency of the electrodes recordings, the extracted SNR values were statistically analyzed and presented using mean as the plotted value and standard deviation as error bars. For each of the three activities and each electrode type, the arithmetic mean of the three SNR values (from Set 1, Set 2, and Set 3) was calculated to represent the typical performance for that specific activity and electrode. The standard deviation (*σ*) of the three SNR values was calculated. This standard deviation was used as the ± error bar to represent the trial‐to‐trial variability (i.e., the consistency) of the SNR measurement within the same session.

### EMG Signal Processing and SNR Comparison

Surface EMG signals were recorded from the left quadriceps femoris muscle group (as shown in Figure 3a) using both conventional Ag/AgCl and hydrogel‐based PPHG electrodes during a single session. Signals were sampled at 200 Hz, while the subject performed controlled motor tasks including sitting, standing, squatting, cyclical movements, and walking.

Raw EMG signals were preprocessed with a bandpass Butterworth filter (20–90 Hz) to isolate muscle activity frequency components and a 50 Hz notch filter to eliminate powerline interference. Cardiac interference was removed by detecting R‐peaks and applying an amplitude‐scaled template subtraction method. The cleaned signals were further denoised using wavelet thresholding with the Daubechies 4 wavelet.

Analysis focused on comparing the preprocessed EMG signals across electrode types. Signal‐to‐noise ratio (SNR) was computed for each electrode during different activities by calculating the variance ratio between preprocessed muscle activity segments and baseline noise segments, expressed in decibels (dB)

(3) $$SNR_{\mathrm{dB}\;}=10\mathrm{lo}g_{10\;}\left(\frac{\sigma_{\mathrm{signal}}^{2}}{\sigma_{\mathrm{noise}}^{2}}\right)$$

where ​σ^2^ denotes variance. Both SNR values and time‐domain preprocessed signals were visually compared to evaluate differences in signal quality between the Ag/AgCl and PPHG electrodes.

### EEG Signal Acquisition and Analysis

Frontal EEG signals were recorded from scalp electrodes positioned at Fp1 and Fp2, referenced to a site behind the right ear, using both conventional Ag/AgCl and hydrogel‐based PPHG electrodes during resting state conditions, as illustrated in Figure 3d. Signals were sampled at 200 Hz, while the subject underwent predefined intervals of baseline rest, eyes‐open mental activity, and eyes‐closed rest.

Preprocessing included detrending to remove slow drifts, followed by low pass filtering at 49 Hz. The cleaned EEG signals were segmented according to the experimental conditions, and power spectral densities (PSD) were computed using Welch's method with 2‐s Hamming windows and 50% overlap. Delta band power (0.5–4 Hz) was extracted from the PSD estimates for each epoch. Temporal changes in delta power were further analyzed using a sliding window approach with 2‐s windows and 0.5‐s steps to produce time‐resolved delta power profiles.

To visualize the EEG's spectral and temporal dynamics and compare electrode performance, continuous wavelet transforms (scalograms) and spectrograms were generated. These analyses provided a comprehensive assessment of the EEG features without formal statistical testing.

### ECG Cohort Study Protocol and Recording Settings

In a silent room, with controlled ambient conditions of 22–23 °C and 48% relative humidity. The participant is seated on a chair and their ECG recorded at rest mostly silent as some participants talked brief periods during the recording. The recording device (ECG‐ON, Khawaja Medical Technology GmBH, Germany) was programmed to record raw signals at 200 Hz. The skin of the participant is prepared by wiping the chest area of recording with an alcohol swipe and is allowed to dry for few seconds. Next, three PPHG electrodes are attached to the recording device and placed securely on the skin and the recording is started for 7 min, followed by another set of PPHG to ensure precision and minimize batch to batch variation and ECG was recorded for another 7 min. Finally, the skin is cleaned by a wipe to remove any residues of the PPHG off the skin (if any) and Ag/AgCl electrodes are placed and ECG recorded for 7 min. The PPHG electrodes recording were done before the Ag/AgCl to avoid mistaking any skin irritation that is typically present after Ag/AgCl electrodes are removed.

### ECG Preprocessing Pipeline

Our ECG processing pipeline initiates through a series of digital filters tailored to enhance frequency ranges where ECG information resides and simultaneously attenuate frequency ranges related to noise sources. Data sampled at 200 Hz are first low pass filtered through an infinite impulse response (IIR) Butterworth filter of order 4 and cut‐off frequency of 90 Hz. Two narrow‐band IIR Butterworth notch filters of order 2 are applied, at the main powerline noise carrier frequency of 50 Hz and its harmonic at 80 Hz, after observing their consistent occurrence in the data. Furthermore, baseline was removed wandering through a moving average filter with a lookback window of 1 s. To obtain comparative noise and ECG content power ratios from the spectral density of each ECG recording (Figure 4e; and Figure S15, Supporting Information), the above filters in isolation was applied and the effect of each filter individually was examined. The remainder of the analysis shown by Figure 5; and Figures S13–S18 (Supporting Information) utilizes the output of the pipeline, i.e., the result of applying all of the above filters on the input. This protocol was applied on the ECG recording stability test (Figure S15, Supporting Information)

### ECG SNR Estimation

For a representative SNR quality comparison, SNR from one participant (p5) was estimated using a variance‐based approach, consistent with the EOG/EMG analysis. R‐peaks were detected using a Pan–Tompkins‐based algorithm, and noise segments were extracted from 100 ms windows placed halfway between successive QRS complexes, effectively isolating baseline fluctuations while avoiding contamination from physiological waveforms. This method captures the intrinsic noise floor of the signal, but differs from the earlier PSD‐based SNR calculation,^[^ ^5^ ^]^ which incorporated a broader frequency noise, including motion artifacts. SNR values were calculated per lead for both Ag/AgCl and PPHG electrodes and visually inspected alongside cleaned ECG signals to confirm the accuracy of noise localization and signal fidelity.

### Machine Learning Analysis of Heartbeats

PCA^[^ ^57^ ^]^ was applied on a per lead basis and the number of principal components that retains 99% of the variance in the data was kept. As the batched heartbeats have a duration of 121 samples or ≈0.6 s, this reduces the dimensionality to about 10–20 dimensions. On the reduced space, the *k*‐means clustering algorithm was applied; in the case of participant identification, the algorithm with 39 centroids was initialized, whereas in the case of lead identification with 3 centroids. Five implementations of the *k*‐means clustering with different random states were run to obtain different centroid initializations, and the best result was kept based on the objective of maximizing the squared Euclidean distance between found cluster centroids after 1000 iterations of the *k*‐means algorithm. For visualization, the PCA‐reduced data along with the cluster centroids through *t*‐SNE,^[^ ^58^ ^]^ with a perplexity of 30 using the Euclidean distance were projected to determine the optimal distance of the 2D projection from the actual high‐dimensional manifold (Figure S15, Supporting Information).

### Evaluation of Clustering Performance

As the analysis so far is unsupervised and the *k*‐means clusters cannot be mapped to ground truth classes arbitrarily, the Munkres assignment algorithm^[^ ^59^ ^]^ was used to solve the assignment problem of cluster labels to actual classes. The Munkres assignment is a greedy mapping algorithm that requires a cost matrix; the cost matrix was constructed by assigning a negative cost to each cluster‐class pair each time they co‐occur in a sample. The cost matrix is then used by the algorithm to determine the cluster‐class pairs that will minimize the total cost of the assignment.

By obtaining the cluster assignments map, then supervised machine learning metrics of accuracy score, micro‐f1 and macro‐f1 were calculated. Accuracy scores were calculated as follows

(4) $$Accuracy=\frac{\sum_{i=1}^{N}1\left(y_{i}=\hat{y_{i}}\right)}{N}$$

where *y_i_* is the true label, $\hat{y_{i}}$ is, the predicted label, N is the total number of samples.

Micro‐f1 score was calculated as follows

(5) $$MicroF1=\frac{2\cdot \sum_{i=1}^{N}1\left(y_{i}=\hat{y_{i}}\right)}{N+\sum_{i=1}^{N}1\left(y_{i}=\hat{y_{i}}\right)}$$

Macro‐f1 score was calculated as follows

(6) $$MacroF1=\frac{1}{C}\sum_{c=1}^{C}F1_{c}$$

where each class's f1‐score *F*1_c_ is

(7) $$F1_{c}=\frac{2\cdot TP_{c}}{N+TP_{c}}$$

where *TP*_c_ corresponds to the per‐class true positives, essentially making *F*1_c_ a per‐class micro‐f1 score.

### Waveform Morphology and Similarity Assessment

The peak‐to‐peak heartbeat amplitude was calculated as

(8) $$A_{\mathrm{pp}}=\max\left(\mathrm{Beat}\right)-\min\left(\mathrm{Beat}\right)$$

To find the P‐wave peak amplitude, the extremum was localized before the Q‐wave, using the annotations from the QRS detection algorithm. Similarly, T‐wave peak amplitude was identified as the distance from the Q wave of the extremum after the S wave.

The aligned cross‐correlation between two aligned heartbeat profiles of length *N*  at lag m≥0 is calculated as

(9) $$\hat{R_{\mathrm{bea}t_{1},\mathrm{bea}t_{2}}}\left(m\right)=\sum_{n=0}^{N-m-1}\mathrm{bea}t_{1}\left[n+m\right]\cdot \mathrm{bea}t_{2}\left[n\right]$$

whereas for *m* < 0 

(10) $$\hat{R_{\mathrm{bea}t_{1},\mathrm{bea}t_{2}}}\left(m\right)=\sum_{n=0}^{N+m-1}\mathrm{bea}t_{1}\left[n\right]\cdot \mathrm{bea}t_{2}\left[n-m\right]$$

To obtain a normalized measure, the cross‐correlation value at lag *m* is divided by the square root of the product of the two beats signal energies

(11) $$\hat{R_{\mathrm{bea}t_{1},\mathrm{bea}t_{2},\mathrm{coeff}}}\left(m\right)=\frac{\hat{R_{\mathrm{bea}t_{1},\mathrm{bea}t_{2}}}\left(m\right)}{\sqrt{\hat{R_{\mathrm{bea}t_{1},\mathrm{bea}t_{1}}}\left(0\right)\cdot \hat{R_{\mathrm{bea}t_{2},\mathrm{bea}t_{2}}}\left(0\right)}}$$

The final value that was adopted as the cross‐correlation value stems from taking the maximum over all lags

(12) $$\rho_{\max}=\max_{m}\left(\hat{R_{\mathrm{bea}t_{1},\mathrm{bea}t_{2},\mathrm{coeff}}}\left(m\right)\right)$$

Similarly, Normalized Root Mean Square Error between two aligned heartbeats is calculated as

(13) $$\mathrm{NRMSE}=\frac{\sqrt{\frac{1}{N}\sum_{i=1}^{N}{\left(\mathrm{bea}t_{1,i}-\mathrm{bea}t_{2,i}\right)}^{2}}}{\max\left(\mathrm{bea}t_{1},\mathrm{bea}t_{2}\right)-\min\left(\mathrm{bea}t_{1},\mathrm{bea}t_{2}\right)}$$

### Experiments

In the signal processing and machine learning analysis of ECG recordings from the PPHG and AgCl sensors, a large‐scale analysis was conducted utilizing the entirety of the available data (Figure 5; and Figures S13–S15, Supporting Information). Out of the initial 43 participants, 3 were excluded because of missing data, and one was excluded on the basis of bad quality data. Individual leads are occasionally discarded by the preprocessing and quality assurance pipeline, nevertheless, the final dataset contains data from all 39 remaining participants. Most of the participants have two PPHG recordings; after initial preprocessing, ECG segments for participants with two PPHG recordings are grouped and processed as a single PPHG recording, as analysis is on a heartbeat level.

To eliminate any systematic bias that the automatic preprocessing pipeline may bear, a second small‐scale analysis was also conducted on four participants, p1, p5, p10, and p39 (Figure 4c–e; and Figures S2 and S17, Supporting Information). One of the authors manually annotated motion artifacts for the recordings of these participants and parts that were annotated as noise were completely removed. Then the preprocessing was applied on the already cleaned data and repeated the power spectral density analysis and per participant heartbeat profile extraction adjusting parameters as needed, e.g., number of clusters equal to 4.

### Ethical Statement

This study was reviewed and approved by the Research Ethics Committee (REC) of Khalifa University of Science and Technology, United Arab Emirates, under protocol number H24‐061. The approval was granted on 11 November 2024 and remains valid until 10 November 2025. The study underwent an expedited review process and was categorized under the collection of biological signals through noninvasive means herein as natural hydrogel electrodes containing PEDOT:PSS, such as ECG and other electrophysiological signals. All participants provided written informed consent prior to inclusion in the study, in accordance with the Declaration of Helsinki and institutional ethical standards. Large language models were used, including ChatGPT (OpenAI) and Claude (Anthropic), to correct the grammar and wording of the text. All text in the manuscript was written by the authors, and large language models were used solely for style corrections with no input in the content or meaning.

## Conflict of Interest

N.B.A, A.M.P, C.P, and A.H.K are co‐inventors of a published, pending U.S. patent application titled “Thermo‐reversible conducting hydrogels and their use for epidermal electrodes or standalone transmitter” (USPTO Application No. 18/621,537, published July 3, 2025). The patent covers materials and processes related to the hydrogel electrode technology described in this study and is under evaluation for potential commercialization. All other authors declare no competing interests.

## Author Contributions

N.B.A. and I.N.Z. contributed equally to the core scientific findings of this work. A.M.P., N.B.A., C.P., and A.K. conceptualized the work. N.B.A., S.R.A., M.A.A., S.B.A., and A.R. performed the electrophysiology experiments. N.B.A. performed and analyzed the electrochemical and dielectric data. I.N.Z. and A.R. conducted the signal processing, N.B.A. conducted a visual inspection and annotation of the ECG data. I.N.Z. conducted the machine learning analysis. N.B.A., I.N.Z., A.R., and A.M.P. wrote the paper.

## Supporting information



Supporting Information

Supplemental Video 1

Supplemental Video 2

## Data Availability

Signal processing, machine learning, and other analysis were performed by means of custom‐made MATLAB scripts. Visualizations were drawn with custom made R scripts. All code regarding the signal processing and machine learning analysis and their products are available at https://github.com/GiannisZgs/Golde_HG_SP_ML_Quality.
