# Supplementary material for: Systematic Benchmarking of a Noise‐Tolerant Conductive Hydrogel Electrode for Epidermal Bioelectronics
Source: Adv Sci (Weinh). 2025 Nov 20;13(20):e15131. doi: 10.1002/advs.202515131 (PMC13067809; doi:10.1002/advs.202515131)
Supplement: Supplementary file 1 — Supporting Information [file ADVS-13-e15131-s002.pdf]

Supporting Information

Systematic Benchmarking of a Noise-free Conductive Hydrogel electrode for Epidermal Bioelectronics

Nazmi B. Alsaafeen[a,b], Ioannis N. Ziogas[a], Shirina R. Alsaedi[a], Mouza A. Alshehhi[a] , Shahd B. Almheiri[a], Adil Rehman[c], Hani Saleh[c], Ahsan Khandoker[a,d], Charalampos Pitsalidis[e,f,g] Antoun Khawaja[h*] Anna-Maria Pappa[a,b,g,i*]*

[a] Department of Biomedical Engineering, Khalifa University, Abu Dhabi, UAE

[b] Center for Separations and Catalysis, Khalifa University, 127788, Abu Dhabi, United Arab

[c] Department of Computer and Information Engineering Khalifa University, 127788, Abu Dhabi, United Arab Emirates

[d] Healthcare Engineering Innovation Center, Khalifa University, 127788, Abu Dhabi, United Arab Emirates

[e] Department of Physics, Khalifa University, 127788, Abu Dhabi, United Arab

[f] Advanced Research and Innovation Center (ARIC), Khalifa University, 127788, Abu Dhabi, United Arab Emirates

[g] Institute of Electronic Structure and Laser (IESL), Foundation for Research and Technology (FORTH), N. Plastira 100, Vassilika Vouton, 70013 Heraklion, Crete, Greece

[h] Khawaja Medical Technologies Gmbh, Germany

[i] Center for Biotechnology, Khalifa University, 127788, Abu Dhabi, United Arab Emirates

*co-corresponding

Keywords: ECG monitoring; conducting hydrogels; wearable electronics; PEDOT:PSS ; conducting polymers; electrophysiological recordings.

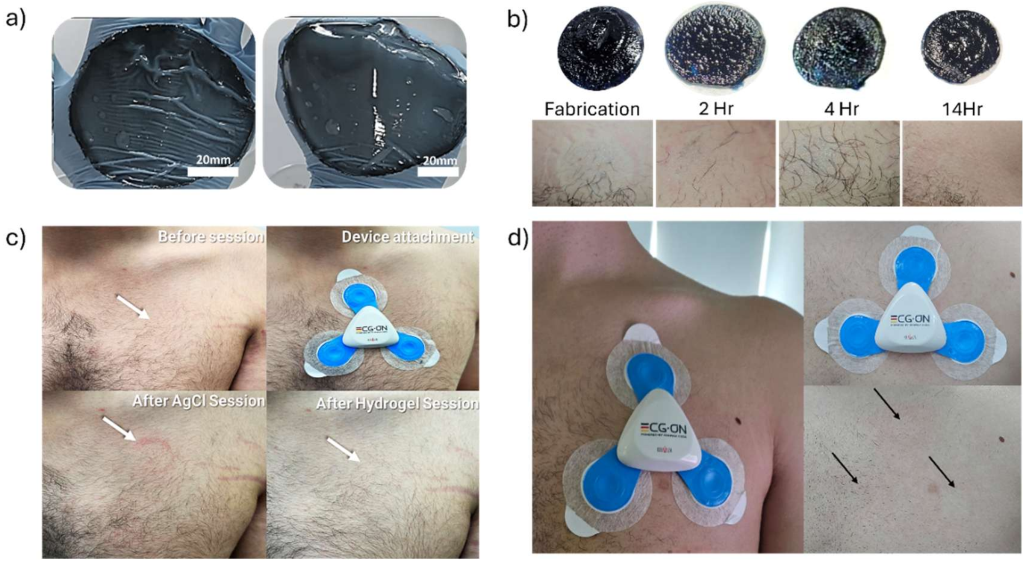

Figure S1: (a) Demonstration of the hydrogel electrode adhesion and stretchability as it adapts to the hand flexion and extension. (b) Hydrogel electrode stability from fabrication, 2hr , 4hr and 14 hr of use and the skin response after each time interval. (c) Skin irritation on a hairy male subject before and after the ECG recording session of each the hydrogel and the Ag/AgCl electrode. (d) Skin irritation on a non-hairy/shaved male subject after only a hydrogel electrode ECG recording session.

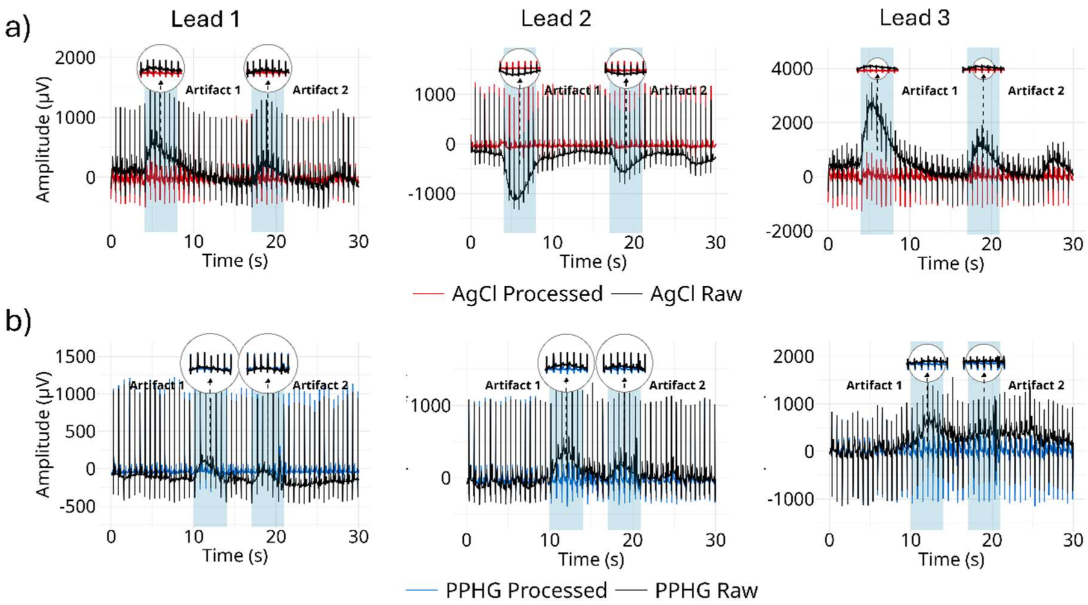

Figure S2: Example of moving artifacts manifestation in 30 seconds of three-lead ECG recording from participant p5. a) AgCl sensor. b) PPHG sensor. This segment has not been manually cleaned for artifacts and is a product of our automatic pre-processing pipeline.

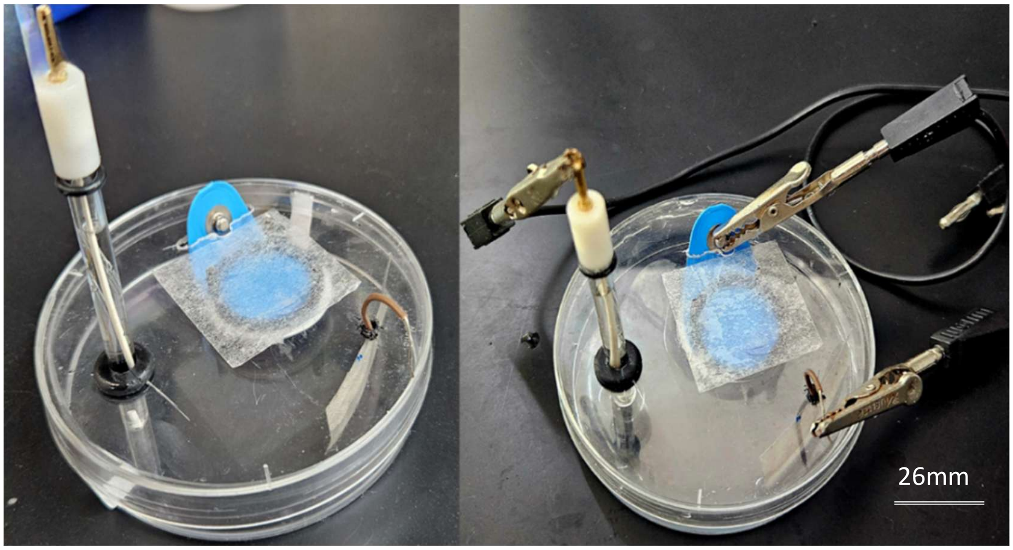

Figure S3: The EIS and CV setup, employing a three-electrode configuration with PBS as the electrolyte. The electrodes were arranged equidistantly with ~26 mm spacing, comparable to standard commercial EIS cells[60]. The active electrode was the ECG electrode, the counter electrode a stainless-steel mesh (twice the active area), and a standard Ag/AgCl electrode served as reference. Each electrode variant was measured three times (n = 3) to minimize sample-to-sample variation.

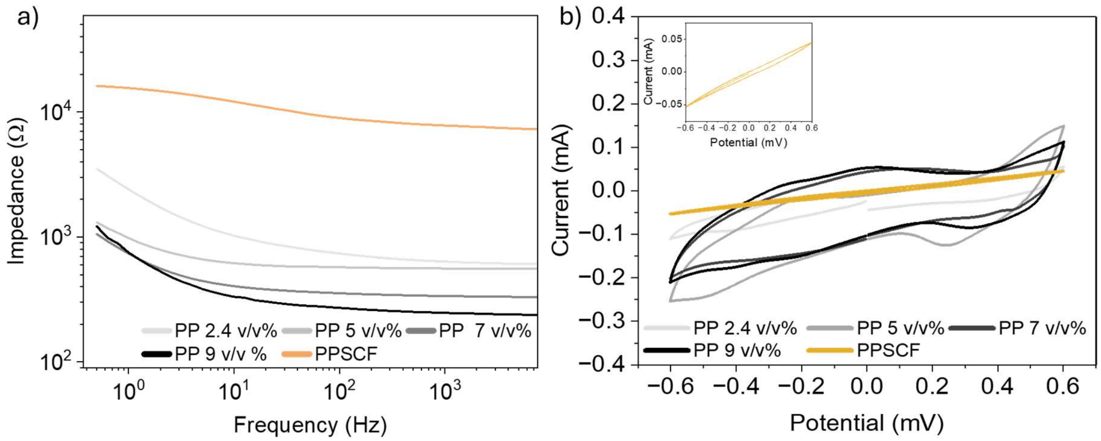

Figure S4: (a) Bode plot comparison of the different doping concentration of PP into the hydrogel electrode against the PPSCF (PP scaffold infused with hydrogel). (b) Comparison of the CV response and area of the CV curves of the different doping concentration of PP into the hydrogel electrode against the PPSCF.

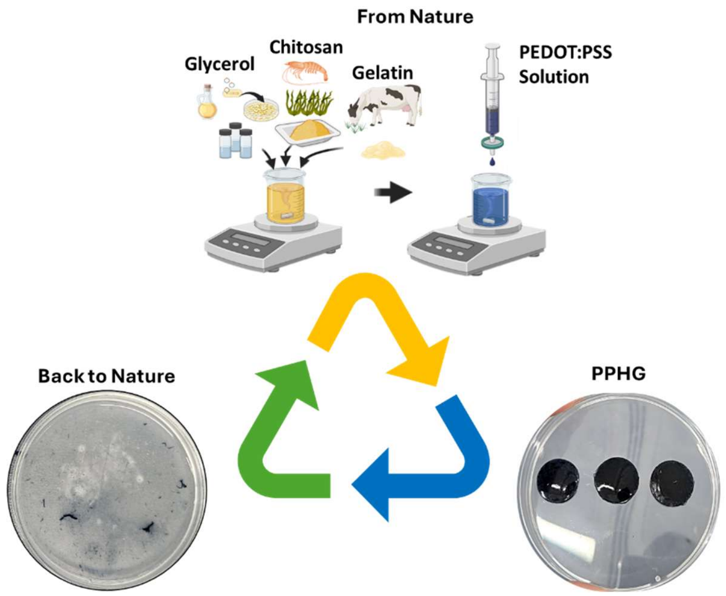

Figure S5: Lifecycle of the hydrogel: raw material sources, fabrication, usage, and degradation after five days in deionized water at indoor conditions.

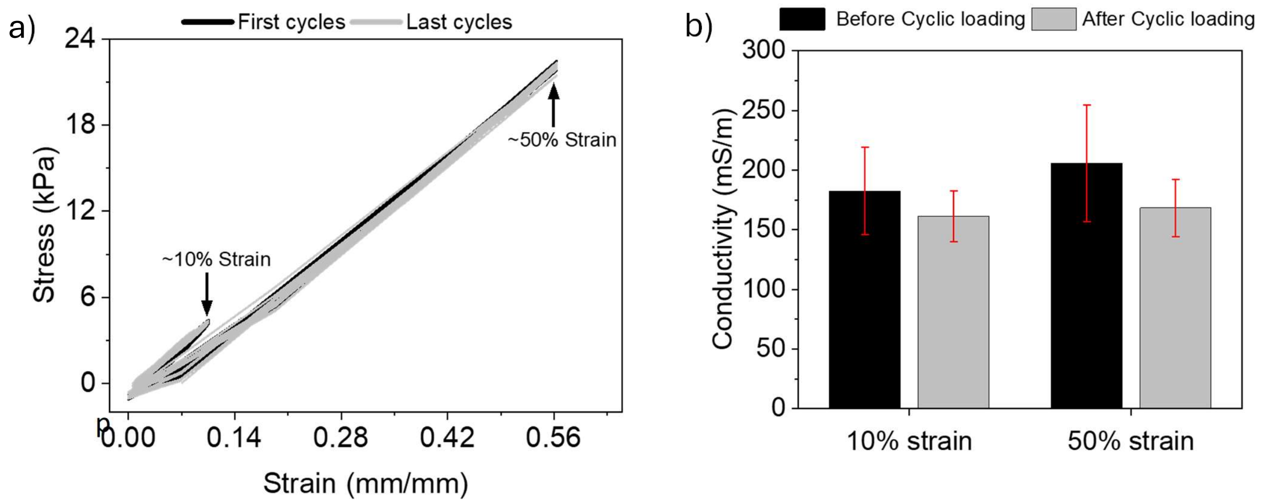

Figure S6: Controlled functional durability testing of PPHG (a) Cyclic mechanical testing showing stable stress–strain response and low hysteresis at physiologically relevant cyclic deformation (10% strain, during a normal breathing cycle, 0.3 Hz) and above typical chest-motion strain (>20% strain, 0.3 Hz).(b) Sheet conductivity at 0 cycles and after 100 cycles. Measurements are the average of n = 3 samples; for the electrical properties, each of the three samples was probed at three different areas of the specimen, yielding nine values for 0 cycles and 100 cycles.

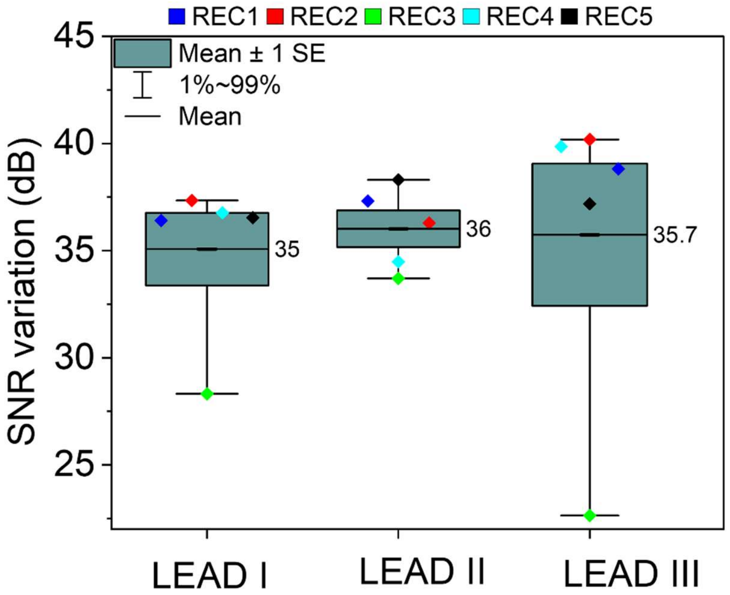

Figure S7: A representative snapshot of a three-lead ECG recording. PPHG electrode samples tested for reusability by taking five separate ECG recording sessions, like the cohort study protocol. ECG recording was taken after four days of storage exposed to indoor conditions.

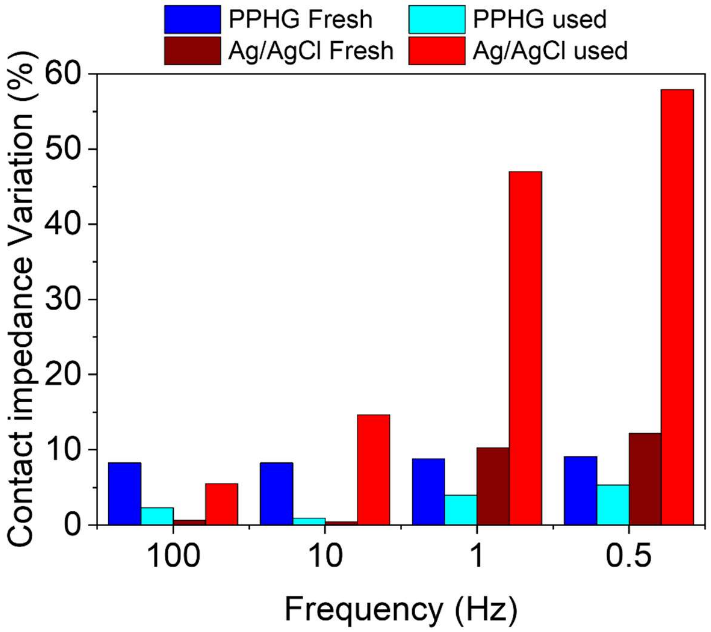

Figure S8: Coefficient of variation (CV) of skin–electrode impedance for PPHG (fresh and used) and Ag/AgCl (fresh and used) electrodes across 0.5–100 Hz. Each bar represents the **coefficient of variation** (%CV = SD/mean × 100) derived from three repeated measurements per electrode condition. Wherever the term "used" is adopted it refers to 5 cycles of wearing and removing the ECG electrode. *Because %CV quantifies relative variability within each electrode type, error bars are not applicable.*

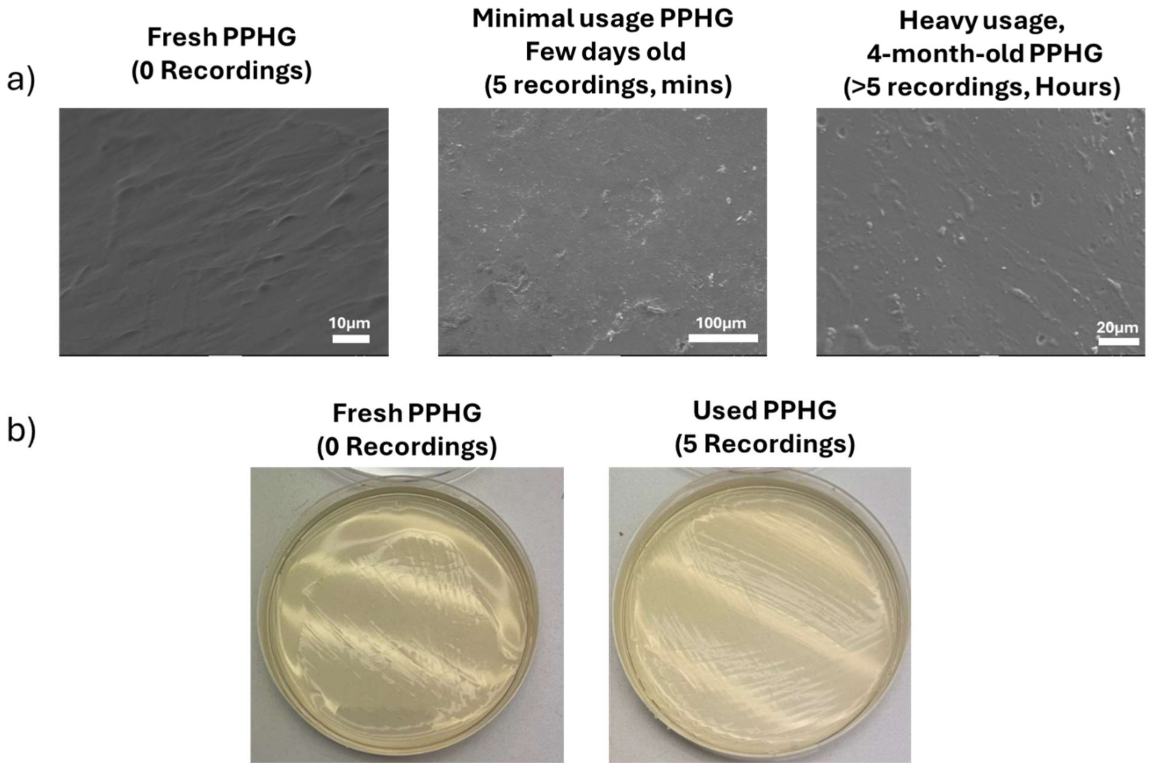

Figure S9: Reusability and biofouling assessment, a) Surface degradation assessment, SEM images of the PPHG surface at various states of usages and storage periods. b) Surface swab bacterial test. Agar plate test showing the absence of bacterial growth on electrodes after repeated use. Swabs were taken from three electrodes before use (0 uses) and after six uses. After incubation for 48 h, no bacterial colonies were observed, confirming that the electrodes remained free of contamination.

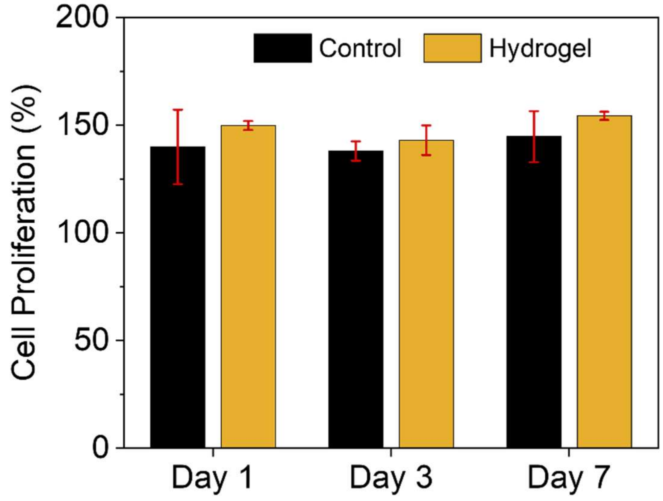

Figure S10: Alamar Blue Assay performed on the hydrogel against PBS media as a control for 7 days to verify the material's biocompatibility.

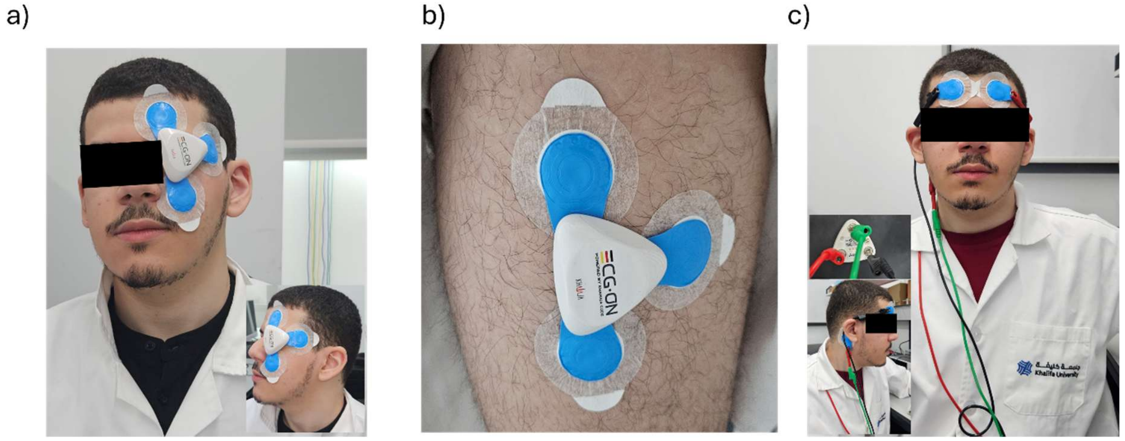

Figure S11: (a) Left eye EOG placement and recording setup of the electrodes and recording device, arranged to detect only vertical eye movements and blinking. (b) Quadriceps femoris EMG signal recording placement of the electrodes and recording device. (c) Frontal EEG electrodes placement and connection with the recording device.

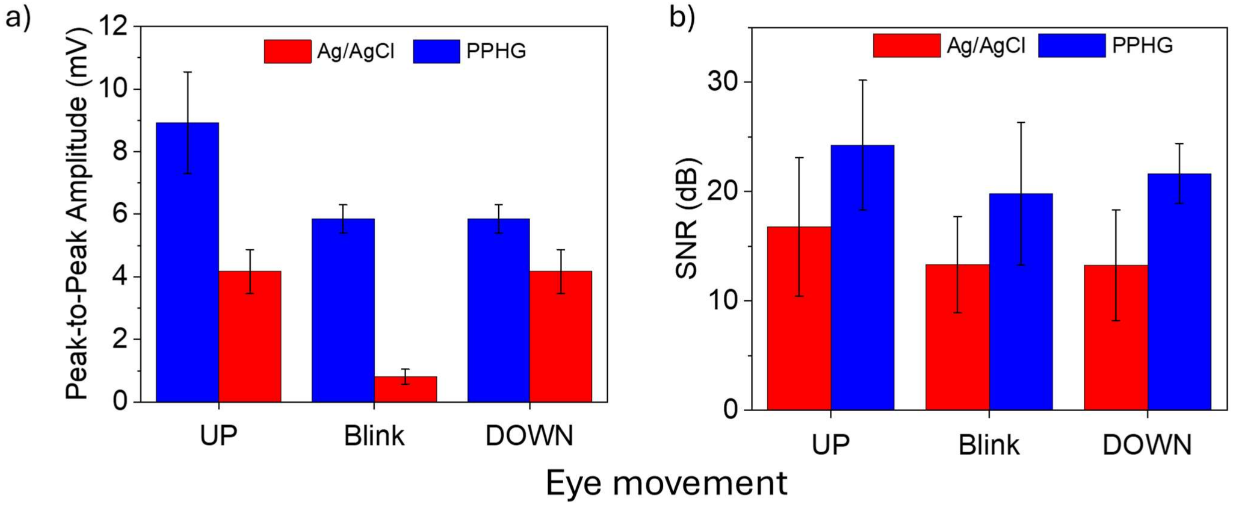

Figure S12: Quantitative comparison of signal amplitude and signal-to-noise ratio (SNR) between conventional Ag/AgCl electrodes (red) and the proposed hydrogel-based PPHG electrodes (blue) during ocular activities including upward gaze, triple blinking, and downward gaze. (a) Mean peak-to-peak amplitude (mV) with standard deviation error bars shows that the PPHG electrode consistently records higher signal amplitudes across all activities. (b) Corresponding SNR values (dB) demonstrate that the PPHG electrode achieves superior noise rejection, with SNR improvements of approximately 1.5 to 2 times compared to Ag/AgCl electrodes, highlighting enhanced signal fidelity during dynamic eye movements.

Processed-to-raw Power Spectral Density Ratio Calculation

We first normalize the input raw and filtered at some frequency range ECG recordings to exclude amplitude effects:

$$x_{\text{norm}} = \frac{x - \mu}{\sigma}$$

where μ is the mean value of x and σ denotes the standard deviation.

We then compute a non-parametric one-sided biased estimate of the power spectral density (PSD) as the Fourier transform of the autocorrelation, for the raw and processed versions of the ECG recording:

$$P(f) = \frac{1}{Nf_s}\left|\sum_{n=0}^{N-1} x[n] \cdot e^{-2\pi if \ \ /f_s}\right|^2$$

To obtain total power of each recording, we calculate the integral (area under the curve) of the periodogram function across the frequency domain axis:

$$P_{total} = \int_0^{f_{max}} P(f)\ df$$

Subsequently, for each frequency range of interest we integrate PSD over that range to obtain a local frequency power:

$$P_{band} = \int_{f_{low}}^{f_{high}} P(f) df$$

The ratio of the PSD as calculated here for the raw and processed sensor data, is used to facilitate comparisons in our analysis (Fig. 4e, Fig. S8).

ECG Quality Assessment Heartbeat Profiling through Forced-Alignment of R-peaks

We use the Pan-Tompkins QRS detection algorithm [116] to obtain annotations of ECG landmarks. Following, we apply an impedance threshold per lead, on two-second segments, to discard signal segments with an impedance higher than 100 Ω. For the R-peak force alignment, we process every R-peak detected by Pan-Tompkins separately; we define fixed search windows around the R-peak to find the P-wave and T-wave landmarks. We allow a window of 250 ms for P-wave and 350ms for T-wave. On the now fixed-length segments that contain the PQRST complex, we apply a variance threshold criterion to exclude highly abnormal heartbeats, artifacts or absence of signal that survived the previous processing steps; we keep heartbeats that lie inside the 1st-95th percentile of the heartbeat variance distribution. The remaining heartbeats constitute the dataset that will be used for the machine learning analysis and is organized into batched heartbeats of identical duration per sensor, per lead and per participant.

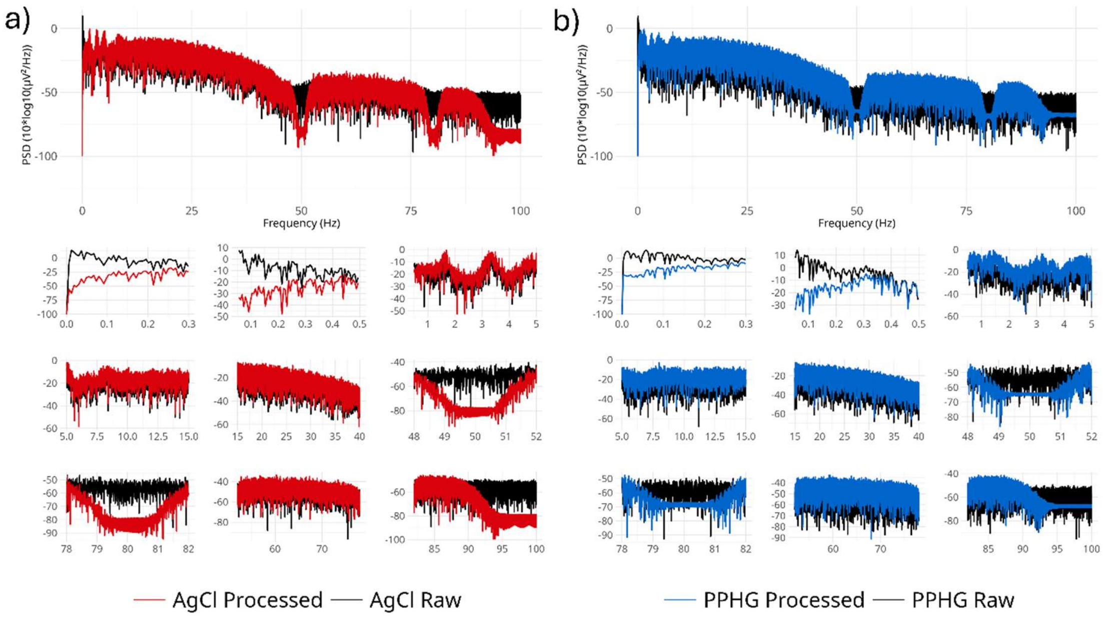

Figure S13: Log-power spectral densities from Lead 1 of manually cleaned data participant p39 (top panels) for the raw data (in black) and the processed data (red and blue) . In the bottom panels, nine different sub-bands of interest are displayed: Baseline Wandering (0-0.3 Hz), Very Low Frequency (0.05-0.5 Hz), Low Frequency (0.5 - 5 Hz), Mid Frequency (5-15 Hz), High Frequency (15-40 Hz), Powerline Noise (48-52 Hz), Ultra High Frequency 1 (52-78 Hz), Powerline Noise Harmonic (78-82 Hz), Ultra High Frequency 2 (82-100 Hz). a) AgCl sensor. b) PPHG sensor.

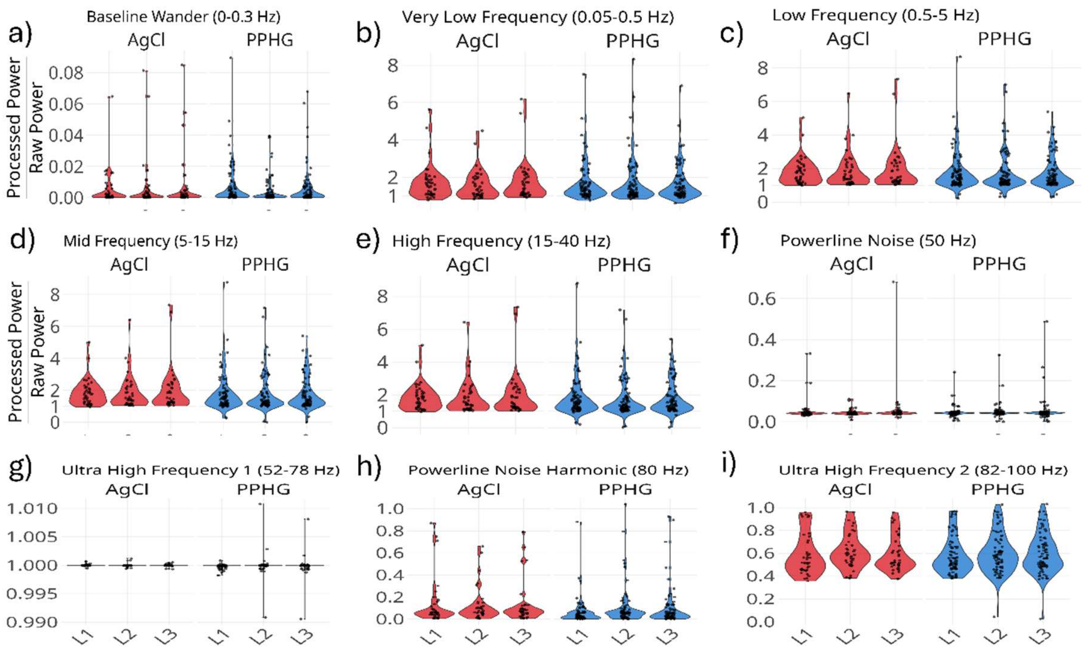

Figure S14: Violin distributions of processed-to-raw power ratio of the ECG in the nine frequency ranges of interest related to noise and ECG content for AgCl (red) and PPHG (blue).

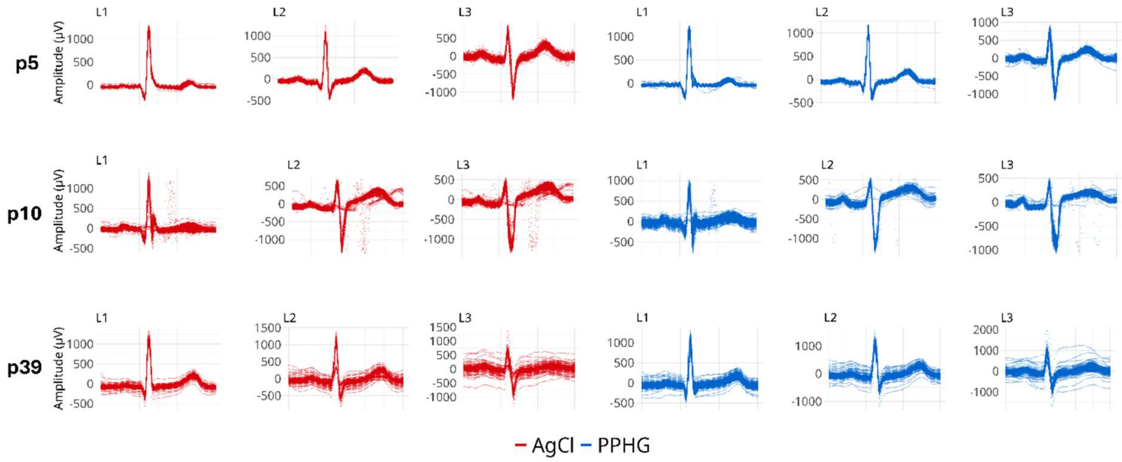

Figure S15: Aligned and overlaid raw heartbeat inputs to the PCA-kmeans-t-SNE clustering and visualization pipeline, per participant, sensor and lead.

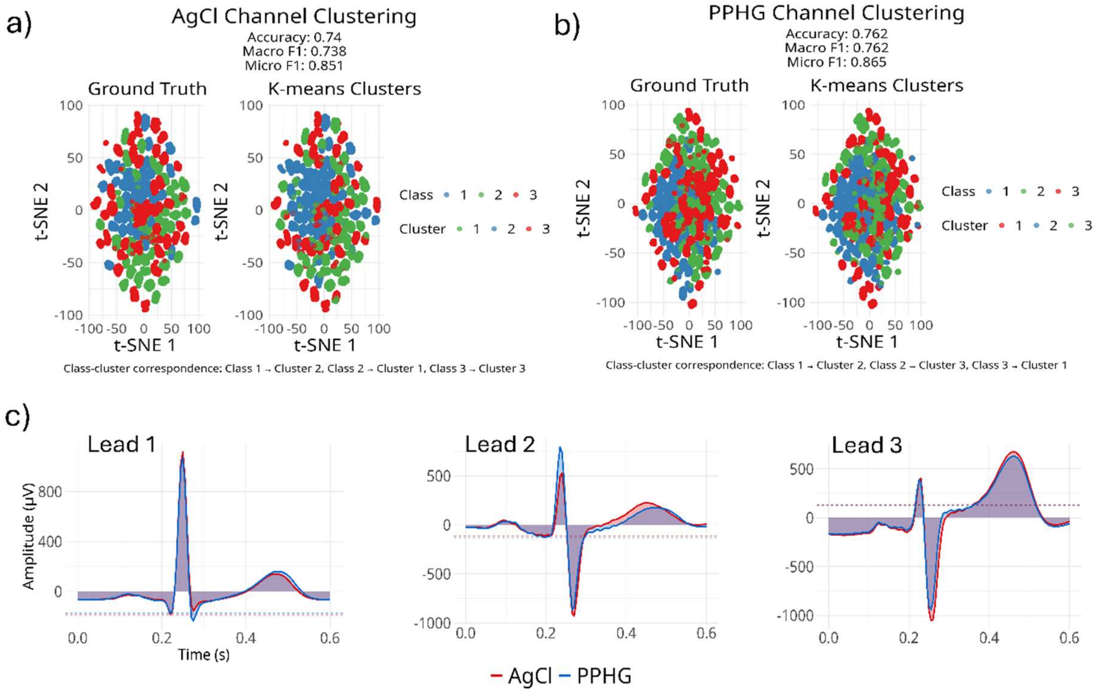

Figure S16: Performance of the unsupervised clustering pipeline in channel identification per sensor. a) AgCl, b) PPHG, c) Inverse PCA transform of the identified centroids to obtain characteristic heartbeats per lead. K-means cluster assignments are mapped to ground truth classes by solving the assignment optimization problem with the Hungarian Munkres assignment algorithm[115]. Accuracy, micro-F1 and macro-F1 scores are measured using the mapped cluster assignments as predictions.

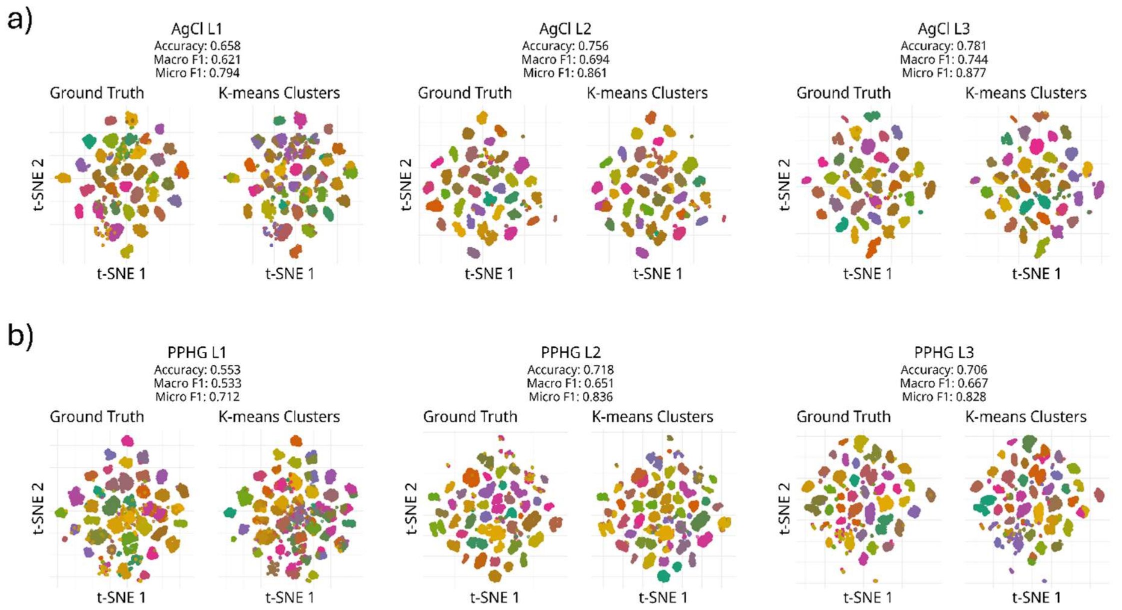

Figure S17 Performance of the unsupervised clustering pipeline in participant identification per sensor and lead. a) AgCl, b) PPHG. K-means cluster assignments are mapped to ground truth classes by solving the assignment optimization problem with the Hungarian Munkres assignment algorithm. Accuracy, micro-F1 and macro-F1 scores are measured using the mapped cluster assignments as predictions.

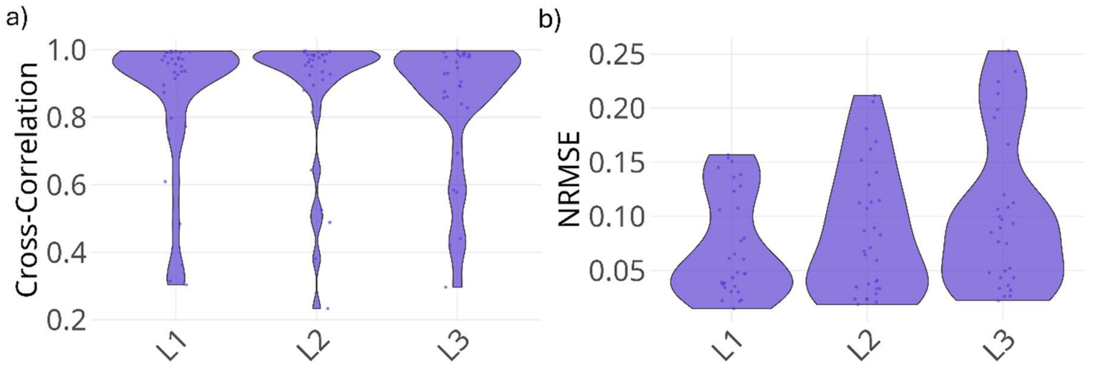

Figure S18: Similarity assessment of the waveform centroids obtained through the inverse PCA transform from the unsupervised clustering using participants as clusters. a)Cross-correlation between the AgCl centroid and PPHG centroid of the same participant and lead. b) Normalized Root Mean Square Error (NRMSE) between the AgCl centroid and PPHG centroid of the same participant and lead.

Table 1: Unweighted Pugh Matrix

	Electrode			Score (1-4)		
	**Ag/AgCl**	**PPSCF**	**PPHG**	**Ag/AgCl**	**PPSCF**	**PPHG**
Signal Quality	Lower bulk impedance (Fig. 2c) Lower frequency capacitance (Fig.2f, 2h)	Lower Skin contact impedance (Fig 2d), Higher dielectric const. (Fig. 2g)	Higher SNR (Fig.3), superior signal fidelity (Fig. 4c-4e). Rapid/smooth polarization (Fig. 2f), Lowest Loss tan δ (Fig. 2h)	3	2	4
Cost	\$0.42, (retail) (Table 3)	\$0.4 (materials) (Table 3,4)	\$0.27 (materials) (Table 3, 4)	2	1	4
Stability	Electrolyte drying affects skin-electrode interface, reducing consistency over time. (Fig 1c, S8)	PEDOT:PSS scaffold fragile. Integration with the hydrogel phase needs improvement. (Fig 1c).	Electrolyte remains stable long-term, maintaining electrode mechanical & electrical stability. (Fig. 1c, S1b, S6- S9)	3	1	4
Reusability	Electrolyte gel smudges the skin, dries over time. Adhesive weakens if reused (2-3 times).	Poor material phase integration with the hydrogel phase causes lower electrical and mechanical performance. (Fig 1c).	Hydrogel's inherent adhesion and homogenous phase, permits higher reliability and multiple uses; consistent performance (Fig. S6-S9).	2	2	4
Ergonomics	Biocompatible, breathable, soft, flexible. Long term application causes discomfort and pain/residue upon removal.	Biocompatible, soft and complaint.	Soft, elastic, skin friendly; provides secure attachment and positive user feedback (Fig. 4g–j, S1b–d).	3	2	4
Environmental friendliness (LOW/MED/HIGH)	LOW, Synthetic materials, metals, high energy & emissions fabrication. Non-biodegradable	MED, mostly synthetic composition, more complex fabrication, moderate biodegradation	HIGH, mostly bioderived, simple & green fabrication, mostly biodegradable.(Fig. 1a, S5)	1	2	4
**Final Score**	**PPHG consistently outperforms across signal quality, stability, cost, reusability, and sustainability. PPSCF shows mechanical fragility and complex fabrication. Ag/AgCl remains a stable benchmark but underperforms in long-term usability and eco-impact.**			**14**	**10**	**24**

Table 2: Summary of sourcing, processing, and disposal characteristics of hydrogel component

Component	**Sourcing**	**Processing**	**Disposal**
Chitosan	Derived from chitin in crustacean shells or fungal cell walls. Renewable and bio-derived via deacetylation of chitin [38].	Hydrophilic in acidic media due to protonated amino groups, making it soluble in mild, water-based systems. Commonly processed into hydrogels, aerogels, films, and coatings using aqueous or mild acidic media. Applied in green electronics (e.g., casting, blends) [38].	Biodegradable, biocompatible, and non-toxic [38]. Degrades naturally via enzymatic and microbial action [38]. Can absorb pollutants (gases, metals, dyes) during its life cycle [38]. Approved for safe use in the US, EU, Korea, and Japan, confirming low toxicity [38].
Gelatin	Derived from animal collagen (bones, hides, skins of pigs, cattle, fish, poultry). Renewable, bio-derived, and widely available; alternatives like fish and avian gelatin avoid cultural/religious restrictions [39].	Water-soluble with excellent gel- and film-forming ability. Easily processed in aqueous systems; properties can be tuned by cross-linking (physical, chemical, enzymatic) or blending with other polymers to tune strength and degradation [39].	Biodegradable, biocompatible, and non-toxic. Degrades naturally via hydrolysis and oxidation, allowing safe environmental reintegration [39].

Glycerol	Glycerol is produced at large scale as a co-product of biodiesel through transesterification of plant and animal triglycerides, making it widely available from bio-based feedstocks such as soy, palm oils, and tallow, and it is also naturally present as the glyceride backbone in fats and oils [40] [41]	Fully miscible with water, glycerol acts as a humectant and plasticizer, enabling entirely aqueous and low-temperature hydrogel preparation without organic solvents, and it is commonly used in Gelatin and chitosan hydrogels and films prepared via solution casting or other water-based methods [42].	Readily biodegradable and rapidly metabolized by microbes, glycerol shows low aquatic toxicity in OECD/ECHA assessments, and because it is water-soluble and of low hazard, standard wastewater and biological treatment systems can effectively handle the modest levels typically used in hydrogels [43].
PEDOT:PSS	PEDOT:PSS is a synthetic polymer, derived from petroleum-based monomers (EDOT and styrene sulfonate), and therefore not renewable or bio-derived [44].	Water-dispersible and stable in aqueous solutions, enabling green processing (drop-casting, spin-coating, spray, inkjet) without toxic solvents [44]. Used in nanocomposites; eco-friendly doping with edge-oxidized graphite (via H_2O_2 oxidation) improves conductivity [45].	Not biodegradable, unlike natural polymers[7]. It is biocompatible, exhibits low cytotoxicity, supporting safe use in biomedical and electronic interfaces. Degradation can be improved by blending small amounts with biodegradable carriers (e.g., cellulose, chitosan, gelatin). [45].

* Stability is based on the how well the electrode properties and overall state is preserved overtime, including mechanical integrity, electrolyte presence, skin contact impedance.

Table 3: Cost and Operational Comparison of Electrodes estimate based on the UAE market.

Electrode	Cost per Electrode	CapEx/Overhead	Operational Complexity
**PPHG**	**Raw Materials $0.15**<br>**With Electrode packaging $0.27**	**Lowest capex, minimal complexity, few equipment needed**	**Low, scalable with minimal overhead due to facile fabrication process**
Ambu Blue Sensor L	Market price<br>~$0.42<br>(500 pcs pack size)	N/A<br>(direct procurement, single-use)	Medium, commercial factory setup Multiple different components and processes
PPSCF sponge	Raw materials ~$0.378<br>(more PEDOT:PSS);<br>With electrode packaging ~$0.4	Higher capex several equipment needed<br>& utilities	High, volume-sensitive, wasteful at low throughput

* Costs reflect primarily the raw material costs (e.g., gelatin, chitosan, glycerol) from Sigma-Alrich bought by UAE market suppliers. See more detailed estimation below.

** For Ambu BlueSensor L, the cost per electrode is calculated as (the retail pack price / by the number of electrodes.)

*** For PPHG and PPSCF, estimates include assumptions on material usage.

**** CapEx includes fabrication equipment where applicable (freeze-dryer, pump, hotplate amortization, utilities e.g. water).

***** Operational complexity reflects fabrication steps, scalability, and waste considerations.

Table 4 : Cost of raw materials of the hydrogel electrodes based on the UAE market

Material	Unit Price ($)	Cost per batch	Cost per electrode	Total Electrode cost (USD)
Gelatin (500G)	266	$1.995	$0.075	-
Chitosan (250G)	23	$0.0014	$0.00005	-
Glycerol (1L)	218	$0.654	$0.025	-
PEDOT:PSS solution (1L)	467	$0.954	$0.054	-
Hydrogel (base)	-	$2.650	$0.096	-
Electrode backing/ connector (from Ambu electrode)	-	-	$0.12	-
PPSCF	-	-	$0.258	$0.378
**PPHG**	**-**	**-**	**$0.15**	**$0.27**

* The cost of deionized (DI) water was not included, as it is negligible relative to the other materials and commonly considered a non-cost laboratory consumable. For the Ambu BlueSensor L electrodes, the cost was obtained by dividing pack cost by the number of electrodes. (179$/500pcs), $0.42/electrode. PPSCF cost was estimated by (cost of PEDOT:PSS amount per electrode+base hydrogel cost per electrode, (0.054*3)+0.096)). PPHG cost was estimated by (base hydrogel cost/electrode+PEDOT:PSS). For the hydrogel electrodes, the total electrode cost, the cost of backing and connector layer was estimated at $0.12 per electrode. Batch volume is 20mL , hydrogel electrode volume is 0.8mL.
